# Ligand Side-Chains Control the Formation of an M_8_L_4_ Molecular Barrel: Unveiling Selective Encapsulation and Sequential Separation Properties

**DOI:** 10.1021/acs.inorgchem.6c00394

**Published:** 2026-03-19

**Authors:** Agnieszka Bajer, Venkateswarulu Mangili, Atiqur Rahman, Jack K. Clegg, Artur R. Stefankiewicz

**Affiliations:** † Center for Advanced Technologies, Adam Mickiewicz University, Uniwersytetu Poznańskiego 10, 61-614 Poznań, Poland; ‡ Faculty of Chemistry, Adam Mickiewicz University, Uniwersytetu Poznańskiego 8, Poznań 61-614, Poland; § School of Chemistry and Molecular Biosciences, The University of Queensland, St. Lucia, Queensland 4072, Australia

## Abstract

Inspired by the importance of steric factors in biomolecular self-assembly, we investigated their role in the exclusive coordination-driven self-assembly of a unique cationic M_8_L_4_ barrel (**MB1**) using a sterically reinforced ligand (**L**). Specifically, we employed a flexible tetrapyridylbiphenyl ligand (**L**) functionalized with ethoxy groups to assess their influence on the self-assembly process with *cis*-Pd­(en)­(NO_3_)_2_. Single-crystal structural analysis of **MB1** revealed that four ethoxy groups are oriented outward, while the remaining four project into the barrel cavity, indicating controlled conformational flexibility around the C_phenyl_–C_pyridyl_ bond during assembly. **MB1**, featuring a sterically crowded cavity, is highly selective in aqueous solution for the encapsulation of hazardous polycyclic aromatic hydrocarbons (PAHs), particularly naphthalene and phenanthrene. The higher binding strength of **MB1** toward naphthalene (*K* ≈ 7.3 × 10^2^ M^–1^) and phenanthrene (*K* ≈ 3.8 × 10^2^ M^–1^) enables their isolation from a mixture of PAHs using **MB1** as a molecular separation flask. In contrast, other M_8_L_4_ barrels featuring wider cavities and larger open windows proved inefficient for such separation. **MB1** therefore enables the challenging sequential aqueous extraction of naphthalene and phenanthrene from PAH mixtures with full reusability.

## Graphical abstract

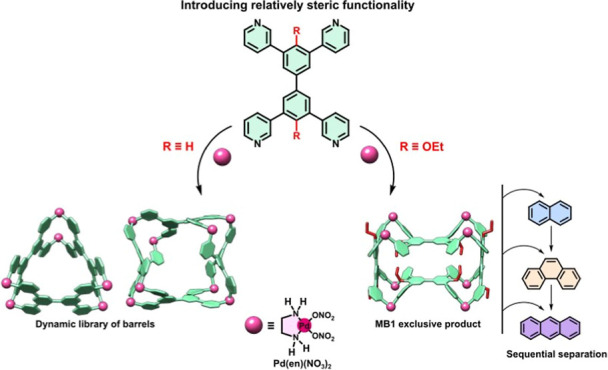

## 1 Introduction

In biological processes, biomolecular self-assembly is an intricate phenomenon that plays a crucial role in diverse mechanisms.^1,2^ Steric interactions between amino acid side chains restrict conformational freedom and enforce specific three-dimensional protein architectures, illustrating how steric demand governs structural organization and function.^3–6^ Inspired by these principles, coordination-driven self-assembly^7–9^ has emerged as a powerful approach for generating discrete structures with predetermined shapes and functionalities.^10–15^

While most donor ligands are designed with rigid backbones to ensure predictable outcomes,^1,16–24^ conformationally flexible ligands often generate dynamic libraries of competing assemblies.^25–29^ In particular, tri- or tetrapyridyl ligands with freely rotating C_phenyl_–C_pyridyl_ bonds can adopt numerous conformers, leading to multiple possible architectures.^30–33^ Although template effects may restrict ligand flexibility,^30,34–36^ achieving precise structural control in the absence of a template remains a significant challenge. Steric modulation represents a promising yet underexplored strategy for regulating ligand conformation and directing the formation of a single, well-defined assembly.^37–44^ Motivated by this knowledge gap, we sought to determine whether steric control of a conformationally flexible tetrapyridyl ligand could enforce the exclusive formation of a well-defined M_8_L_4_ molecular barrel while enabling its application in the selective and stepwise separation of PAHs in aqueous solution.

Precise control over supramolecular geometry is particularly appealing for the selective separation of structurally similar molecules. A representative challenge is the separation of polycyclic aromatic hydrocarbons (PAHs), which exhibit closely related physicochemical properties and are difficult to distinguish using conventional techniques.^45–47^ Importantly, many PAHs are environmentally persistent and associated with mutagenic and carcinogenic effects, underscoring the need for efficient and selective separation strategies.^48–50^ Although methods such as distillation and chromatography are employed industrially, they are often energy-intensive and inefficient for fine discrimination of closely related PAHs.^51–53^ The design of supramolecular systems in which steric factors dictate selective binding or assembly offers a promising strategy to overcome these limitations.

Although several water-soluble cages capable of discriminating between related PAH isomers have been reported,^54–62^ systems enabling stepwise and sequential separation using a single host remain limited.^63,64^ The rational design of new water-soluble hosts with precisely defined cavities and sterically controlled guest recognition is therefore highly desirable.^65^

Herein, we present the design and synthesis of an M_2*n*_L_*n*_-type dumbbell-shaped molecular barrel (**MB1**) formed exclusively from an ethoxy-substituted tetrapyridylbiphenyl ligand (**L**) and *cis*-Pd­(en)­(NO_3_)_2_ (Figure 1). The ethoxy groups were introduced ortho to the pyridyl-phenyl linkage to increase steric hindrance near the rotational axis, thereby restricting C_pyridyl_–C_phenyl_ bond rotation and ligand conformational flexibility, promoting a preorganized geometry that enables directional self-assembly. Ethoxy substituents were selected as an optimal compromise between steric demand and synthetic accessibility. Moreover, these substituents effectively limit conformational flexibility without interfering with Pd–N coordination. The ethoxy substituents adopt both endohedral and exohedral orientations, increasing steric congestion around the rotational axis and reinforcing conformational restriction, thereby promoting the exclusive formation of a well-defined assembly.

**Figure 1:**
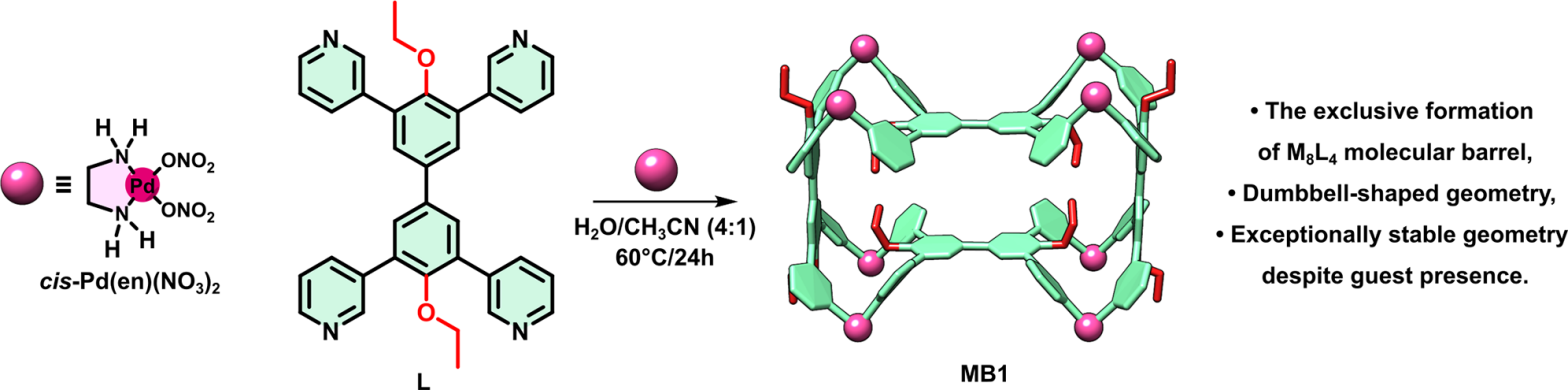
Schematic representation of the self-assembly of the ethoxy-substituted ligand derivative into a single, well-defined coordination cage **MB1**.

Structural analysis revealed that the **MB1** adopted a dumbbell-shaped geometry with wide-open windows and a large interior cavity. In aqueous solution, **MB1** encapsulates planar PAHs, and systematic binding studies demonstrated selective recognition of specific PAHs. Leveraging these distinct binding capabilities, **MB1** enables stepwise separation of a mixture of naphthalene (NAPH), phenanthrene (PHE), and anthracene (ANT). To the best of our knowledge, this represents the first example of a water-soluble molecular barrel capable of sequential PAH separation through simple aqueous extraction within a single supramolecular host.

## 2 Results and Discussion

### 2.1 Self-Assembly of Pd­(II) Barrel

Fujita and co-workers reported the formation of a dynamic library of M_2*n*_L_*n*_-type molecular barrels by reacting the unsubstituted tetrakis­(3-pyridyl)­biphenyl ligand with *cis*-Pd­(en)­(NO_3_)_2_.^33^ When the complexation was performed in the presence of an external template (biphenyl), the system preferentially yielded a templated hexanuclear barrel (trimeric prism). Upon removal of the template, the dynamic metal–ligand bonds underwent reorganization, leading to the formation of a dynamic library of assemblies in solution. Slow evaporation of the solution led to the crystallization of a tetrameric box, representing the least soluble species. A possible interpretation of this structural lability is that there is unrestricted rotation about the peripheral C_phenyl_–C_pyridyl_ bonds of the ligand.

In an effort to obtain a single, well-defined Pd_2*n*_L_*n*_ assembly without relying on external templates, Chand and co-workers^66^ designed a pyrene-core-modified tetrakis­(3-pyridyl)­ligand intended to suppress rotation around the central C–C bond. However, despite the increased rigidity, complexation with *cis*-Pd­(en)­(NO_3_)_2_, both in the presence and absence of a template, produced mixtures of assemblies rather than a single discrete structure. These observations indicate that even when rotational freedom around the central bond is restricted, the remaining flexibility around the peripheral C_phenyl_–C_pyridyl_ bonds is sufficient to generate multiple competing architectures. Overall, these studies underscore the delicate balance between rigidity and flexibility required in ligand design to achieve exclusive assemblies.

Building on these precedents, we aimed to suppress the free rotation of the peripheral C_phenyl_–C_pyridyl_ bonds to synthesize an exclusive Pd_2*n*_L_*n*_-type molecular assembly without the assistance of external templates. To this end, ethoxy substituents were introduced onto the biphenyl core to increase steric demand and suppress flexibility around the C_phenyl_–C_pyridyl_ bond.^32,33,67^ These ethoxy functionalizations induce local steric crowding around the C_phenyl_–C_pyridyl_ bond, limit rotational freedom, and enforce an optimal conformation. This conformational preorganization directs the ligand toward the curvature and geometry required for exclusive formation of the Pd_2*n*_L_*n*_-type molecular assembly.

Complexation of the sterically reinforced ligand (**L**) with *cis*-Pd­(en)­(NO_3_)_2_ in a 2:1 molar ratio at 65 °C in H_2_O/CH_3_CN (4:1) gave a clear, colorless solution. The ^1^H NMR spectrum of the resulting solution displayed a set of ten well-resolved aromatic-H signals ranging from 9.24 to 7.84 ppm with equal integration (Figure 2b), in sharp contrast to the free ligand (**L**), which exhibits just five distinct peaks in the aromatic region (Figure 2a). This, coupled in particular with the very different shifts of two ethoxy-methyl multiples, clearly shows either the presence of equal numbers of two inequivalent ligand units within the complex formed, taken to be a single species based on the uniform peak integrals, or the presence of some species where Pd­(II) coordination has rendered the ligand unsymmetrical. On the basis of known literature,^57^ the first alternative is favored. COSY and NOESY NMR analysis enabled assignment of all proton signals (Figures S5 and S6). All pyridyl protons exhibit downfield shifts, with four of them appearing at 9.83, 9.32, 9.14, and 8.87 ppm, which are distinct from the corresponding ligand **L** protons (Figure 2b).

**Figure 2:**
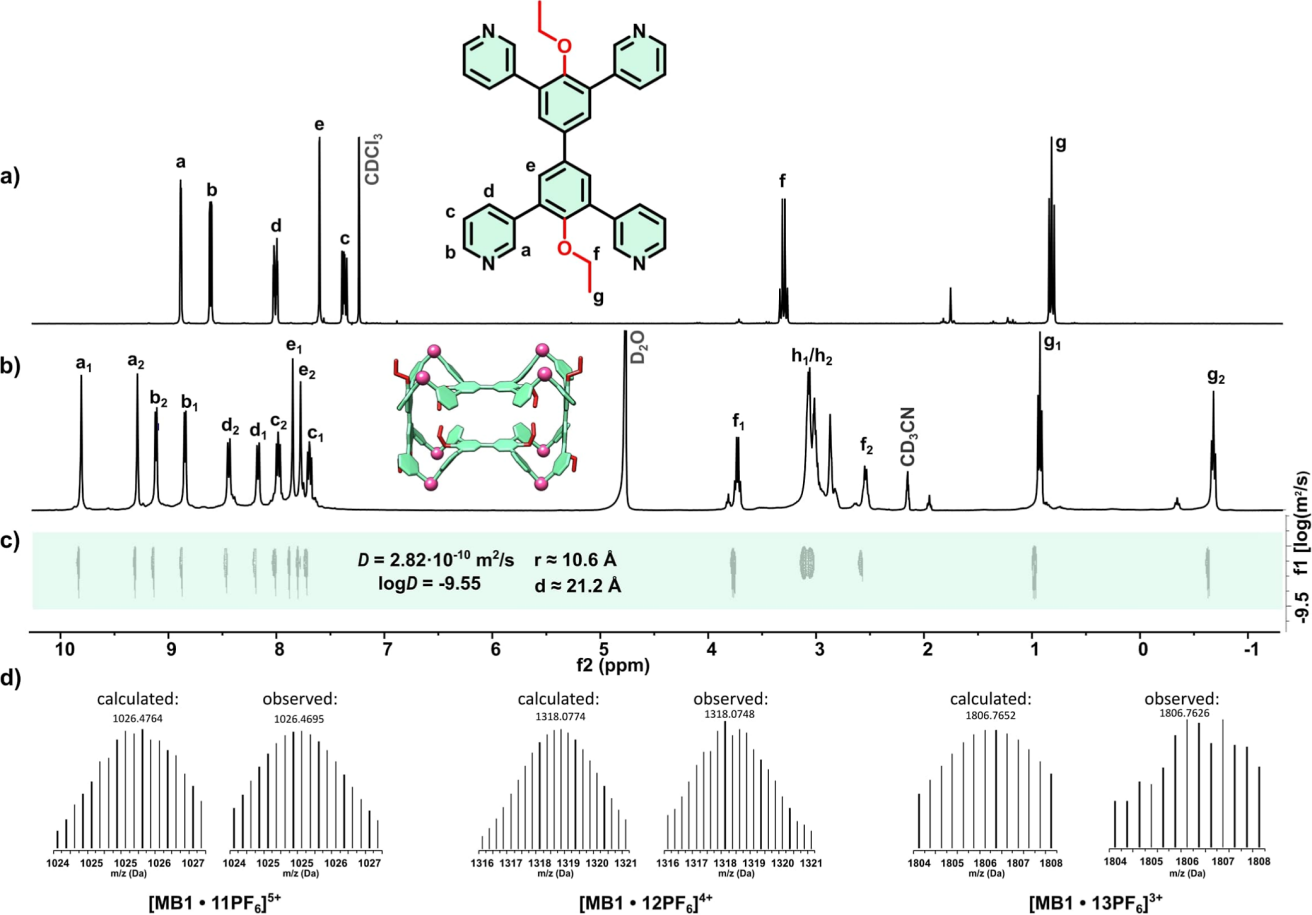
^1^H NMR spectra of (a) ligand **L** in CDCl_3_ (300 MHz); (b) **MB1** in D_2_O/CD_3_CN (4:1 *v*/*v*) (300 MHz); (c) ^1^H DOSY NMR of **MB1** (600 MHz); (d) The ESI-MS spectrum presents the simulated (left) and experimental (right) *m*/*z* pattern of **MB1** (PF_6_^–^ counterions) in acetonitrile.

^1^H DOSY NMR firmly established the formation of a single, large assembly, showing a single band with a diffusion coefficient of a log *D* = −9.55, and the calculated hydrodynamic radius is r ≈ 3.9 Å (Figures 2c, and S7). The corresponding hydrodynamic radius is consistent with the dimensions of the internal cavity observed in the solid-state structure. To gain insight into the structural composition of **MB1**, its PF_6_^–^ salt was prepared by adding an excess of KPF_6_ to the aqueous solution of the synthesized nitrate. The obtained precipitate was isolated and characterized by electrospray ionization mass spectrometry (ESI-MS). The spectrum displayed multiple ion-associate peaks with significant abundances at *m*/*z* = 1806.76, 1318.07, 1025.47, and 830.40, corresponding to [M_8_L_4_•13PF_6_]^3+^, [M_8_L_4_•12PF_6_]^4+^, [M_8_L_4_•11PF_6_]^5+^, and [M_8_L_4_•10PF_6_]^6+^, respectively (Figures 2d, and S8), all consistent with **MB1** having an [8 + 4] stoichiometry.

To better understand the origin of the pyridyl signal splitting observed in solution and to determine the precise geometry of the assembly, diffraction-quality single crystals of **MB1** were obtained by slow diffusion of THF vapors into an aqueous solution of the complex. X-ray diffraction established the structure of **MB1** (Figure 3), which crystallized in a triclinic *P*1 space group, with the asymmetric unit containing half of the assembly -that is, two ligands and four Pd­(II) metal centers. The eight Pd­(II) centers occupy the vertices of a tetrafacial barrel, while the four donor ligands cover the four rectangular walls. A notable structural feature is the distinct orientation of the ligand substituents: in two ligands, both the ethoxy side chains and the pyridine rings adopt endohedral orientations, whereas in the other two ligands they orient exohedrally. This structural asymmetry correlates with the splitting of the pyridyl protons observed in the ^1^H NMR spectrum. Further, to investigate whether the endohedral and exohedral configurations of the ethoxy substituents observed in the solid state persist in solution, variable-temperature (VT) ^1^H NMR studies were performed in D_2_O and D_2_O/CD_3_CN (4:1) over the temperature range of 10–60 °C (Figures S9 and S10). Throughout this range, the resonances of the ethoxy substituents appear as a single set of signals, indicating fast conformational exchange on the NMR time scale. Increasing temperature results in gradual downfield shifts of the corresponding proton signals without peak splitting or coalescence. These findings demonstrate that the two orientations observed crystallographically are dynamically interconvertible in solution. The temperature-dependent chemical shift changes are consistent with variations in solvent interactions and the local electronic environment rather than the presence of discrete static conformers. Thus, the steric regulation inferred from the crystal structure most likely reflects a dynamic equilibrium in solution. Local steric crowding near the biphenyl-pyridyl bond enforces rotation, resulting in a dihedral angle of ∼45° between the pyridine and biphenyl ring planes. Distances between opposite Pd­(II) centers vary depending on their position within the cage. Pd1–Pd3 and Pd2–Pd4 separations are 13.57 Å and 13.50 Å, respectively, whereas adjacent Pd­(II) centers are separated by approximately 13.49 Å and 13.39 Å. The interior hydrophobic cavity is defined by the four aromatic faces of the ligands and spans approximately 8.6 Å in width and 18.5 Å in length. This elongated cavity creates a narrow binding pocket well-suited for hosting planar aromatic hydrophobic guests through π–π stacking. The introduction of ethoxy substituents likely accounts for the formation of a single dumbbell-shaped cation, whereas structurally similar ligands yielded the expected dynamic species.^33,66^

**Figure 3:**
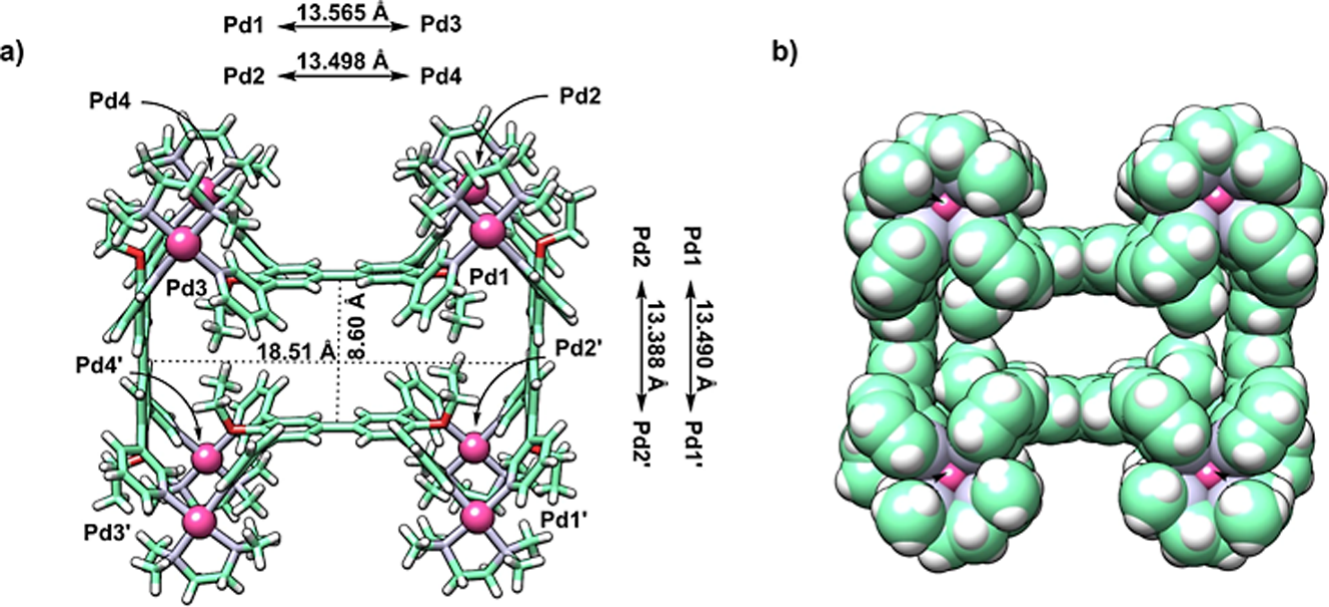
Single crystal structure of **MB1**: (a) Capped-stick representation showing Pd–Pd distances and the distance between ligands in the inner cavity; (b) Space filling model in side view. Solvent molecules and counteranions were omitted for clarity.

### 2.2 Host–Guest Studies

Given its hollow-walled cavity, two wide-open windows, hydrophobic interior, and water solubility, we were prompted to explore the guest-binding capability of **MB1** in aqueous media.^55,58^ To this end, an excess amount (ca. 5 equiv) of various planar polycyclic aromatic hydrocarbons (PAHs) including naphthalene (NAPH), phenanthrene (PHE), anthracene (ANT), pyrene (PYR), perylene (PER), triphenylene (TRIP), and coronene (COR) was added to a solution of **MB1** (2.2 mM) in D_2_O/CD_3_CN (4:1). After stirring the mixtures at room temperature for 24 h, the unbound guests were removed by centrifugation, and the resulting clear solutions of the host–guest complexes were characterized by NMR spectroscopy (Figures 4 and S11). In the ^1^H NMR spectra of NAPH⊂**MB1** and PHE⊂**MB1**, a new set of peaks appeared in the 5–7 ppm region (Figure 4b,c), characteristic of encapsulated aromatic guests.^55,56,68^

**Figure 4:**
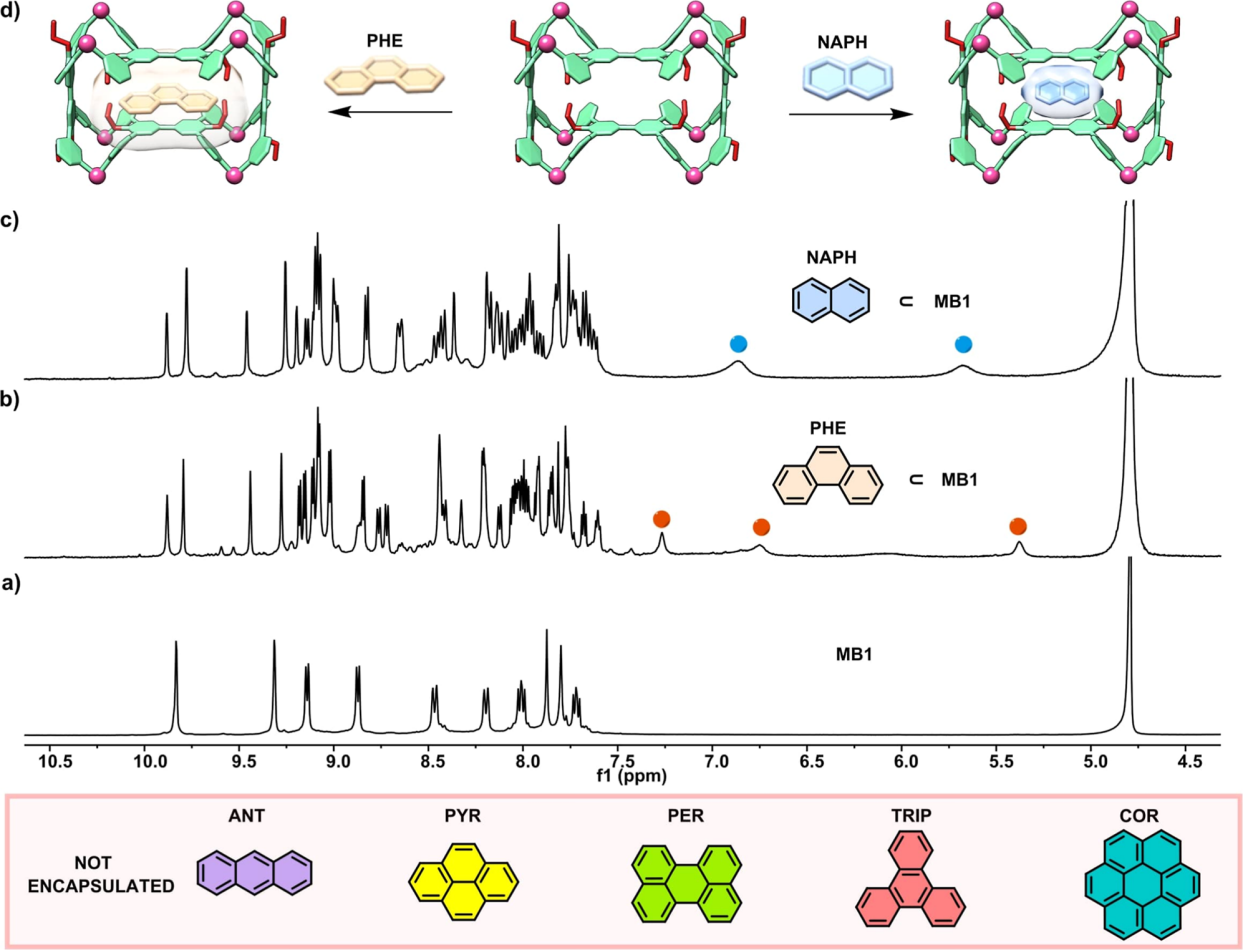
Encapsulation of phenanthrene and naphthalene inside **MB1**: partial ^1^H NMR (300 MHz, D_2_O: CD_3_CN (4:1 v/v)) stack plot of (a) **MB1**, (b) PHE⊂**MB1** (PHE: Phenanthrene), (c) NAPH⊂**MB1** (NAPH: Naphthalene) showing the changes in NMR through guest encapsulation and (d) schematic representation of the encapsulation of phenanthrene and naphthalene. Guest signal peaks are represented by balls (Phenanthrene and Naphthalene). (Inset) Examples of guests that **MB1** was unable to encapsulate.

In the absence of guests, **MB1** exhibits well-resolved sharp signals between 10.5–7.5 ppm in the ^1^H NMR spectrum, corresponding to the pyridyl and biphenyl protons of the barrel. Upon addition of the guest, several of these sharp signals undergo pronounced shifts, reflecting noncovalent CH···π and π···π interactions between the PAH guests and the aromatic pocket of the host. In particular, guest encapsulation led to the doubling of several host proton signals, reflecting shifts in the chemical environment of the host protons indicating a loss of **MB1** symmetry. This desymmetrization is attributed to inequivalent orientations of the encapsulated guest within the cavity. Because PAH guests tend to sit closer to one opening of the dumbbell-shaped barrel,^69^ the ligand protons experience two distinct local environments. This asymmetry in guest positioning commonly produces signal splitting in host–guest systems with restricted internal space.^70^ Additionally, minor signals are observed in the aromatic region, which are most likely arises from alternative orientations of the guest within the host cavity or other minor conformers of the host–guest. Interestingly, in the presence of other PAHs, the ^1^H NMR spectra of **MB1** remained unchanged, and no additional peaks were observed (Figure S23). These results indicate that **MB1** does not possess a sufficiently large cavity to accommodate bulkier guests beyond naphthalene and phenanthrene.

To evaluate the effect of the confined environment on the photophysical properties of the guest molecules, fluorescence spectra of NAPH⊂**MB1** and PHE⊂**MB1** were recorded alongside the empty barrel **MB1** and the free guests under identical conditions (Figure S18). NAPH⊂**MB1** exhibits an emission band at ∼320–340 nm attributable to π–π* transitions of the encapsulated NAPH. Similarly, PHE⊂**MB1** displays emission in the ∼340–380 nm range corresponding to π–π* transitions of encapsulated PHE. In contrast, the free guests show very weak fluorescence in aqueous medium due to their low solubility and aggregation-caused quenching, whereas encapsulation results in an approximately 10-fold enhancement of emission intensity. These observations indicate that the confined hydrophobic environment of the barrel modulates the excited-state behavior of the guests and provides additional support for host–guest complex formation in solution.

Further support for host–guest complex formation was obtained from DOSY NMR spectroscopy. The spectra of both NAPH⊂**MB1** and PHE⊂**MB1** displayed a single diffusion band corresponding to log *D* = −9.65 and −9.63, respectively. Moreover, the calculated hydrodynamic radii (∼3.9 Å for **MB1**, ∼4.9 Å for NAPH⊂**MB1** and ∼4.7 Å for PHE⊂**MB1**) are in good agreement, confirming that the host and guest diffuse together as discrete supramolecular assemblies (Figures S12, and S15). These combined ^1^H NMR and DOSY data confirm that the minor conformers of the host–guest or guest orientations contribute to spectral complexity, the predominant species in solution corresponds to the fully formed host–guest assemblies, confirming the selective encapsulation in solution. Additionally, 2D NMR experiments (COSY and NOESY) provided complementary evidence for encapsulation, showing significant correlations among the guest-derived peaks as well as cross-peaks between the host and guest signals (Figures S13–S17), thereby confirming formation of the host–guest assemblies. To determine the host–guest stoichiometry, ^1^H NMR titration experiments were performed using solutions of the guest (1 mM in CD_3_OD) and **MB1** (4.481 mM in D_2_O) in the absence of acetonitrile. Although the addition of acetonitrile improves the spectral resolution of the **MB1** solution, performing titrations in a D_2_O/CD_3_CN (4:1) mixture together with the guest in CD_3_OD created a ternary solvent system that produced inconsistent chemical shifts and nonreproducible binding isotherms. Conducting all titrations in pure D_2_O eliminated these artifacts and yielded reliable, internally consistent data.

Titrations were done by adding solutions of naphthalene and phenanthrene (1 mM in CD_3_OD) to **MB1** in D_2_O at 25 °C. ^1^H NMR spectra recorded immediately after each addition showed well-resolved, separate host and host–guest peaks, indicating slow exchange on the NMR time scale (Figures S19, and S21). Quantitative integration of the ^1^H NMR signals allowed determination of the concentration of the host–guest complex [HG]. Plotting [HG] against the equivalents of added guest resulted in a saturation at approximately one equivalent, confirming a 1:1 host–guest stoichiometry for both systems. The titration data were fitted to the Hill equation,^71^ using the chemical-shift changes of the most sensitive proton signal of **MB1**. The resulting apparent binding constants were K_a_ = 7.3 × 10^2^ for NAPH⊂**MB1** and *K*_a_ = 3.8 × 10^2^ for PHE ⊂ **MB1** (Table 1, Figures S20, and S22).^72^ To further quantify the thermodynamics of encapsulation, the Gibbs free energies of complex formation were calculated, yielding −Δ*G*° = 16.36 for NAPH⊂**MB1** and 14.72 kJ/mol for PHE⊂**MB1**. These results indicate that the **MB1** exhibits a stronger affinity and selectivity toward naphthalene, consistent with its more favorable fit, optimal molecular size, and stronger hydrophobic interactions within the confined cavity of the barrel.

**Table 1:** Binding Constants for the Complexation of **MB1** with Different Guests as Determined by ^1^H NMR Titration Experiments at 298 K, and Gibbs Free Energies of Host–Guest Assemblies

Guest	*K*_a_ (M^–1^)	–Δ*G*° at 298 K (kJ/mol)
Phenanthrene (PHE)	3.8 × 10^2^	14.72
Naphthalene (NAPH)	7.3 × 10^2^	16.36

### 2.3 Sequential Separation of Naphthalene and Phenanthrene from Anthracene Mixture

The selective encapsulation and distinct binding affinities of **MB1** suggested that it could be employed for the stepwise separation of naphthalene, phenanthrene, and anthracene (Figure 5). All separation experiments were performed exclusively in water due to the irreproducible chemical shifts observed in the presence of organic solvents. To evaluate the possibility, a D_2_O solution of **MB1** was treated with a PAH mixture composed of naphthalene, phenanthrene, and anthracene, each at 2 equiv. relative to the host. After stirring for 24 h, the mixture was centrifuged to separate the aqueous phase containing the host–guest complexes from the unbound solid guests, and characterized. The ^1^H NMR spectrum of the clear supernatant displayed signals corresponding to both NAPH⊂**MB1** and PHE⊂**MB1**, confirming the selective uptake of these two guests, while anthracene remained unbound (Figure S23).

**Figure 5:**
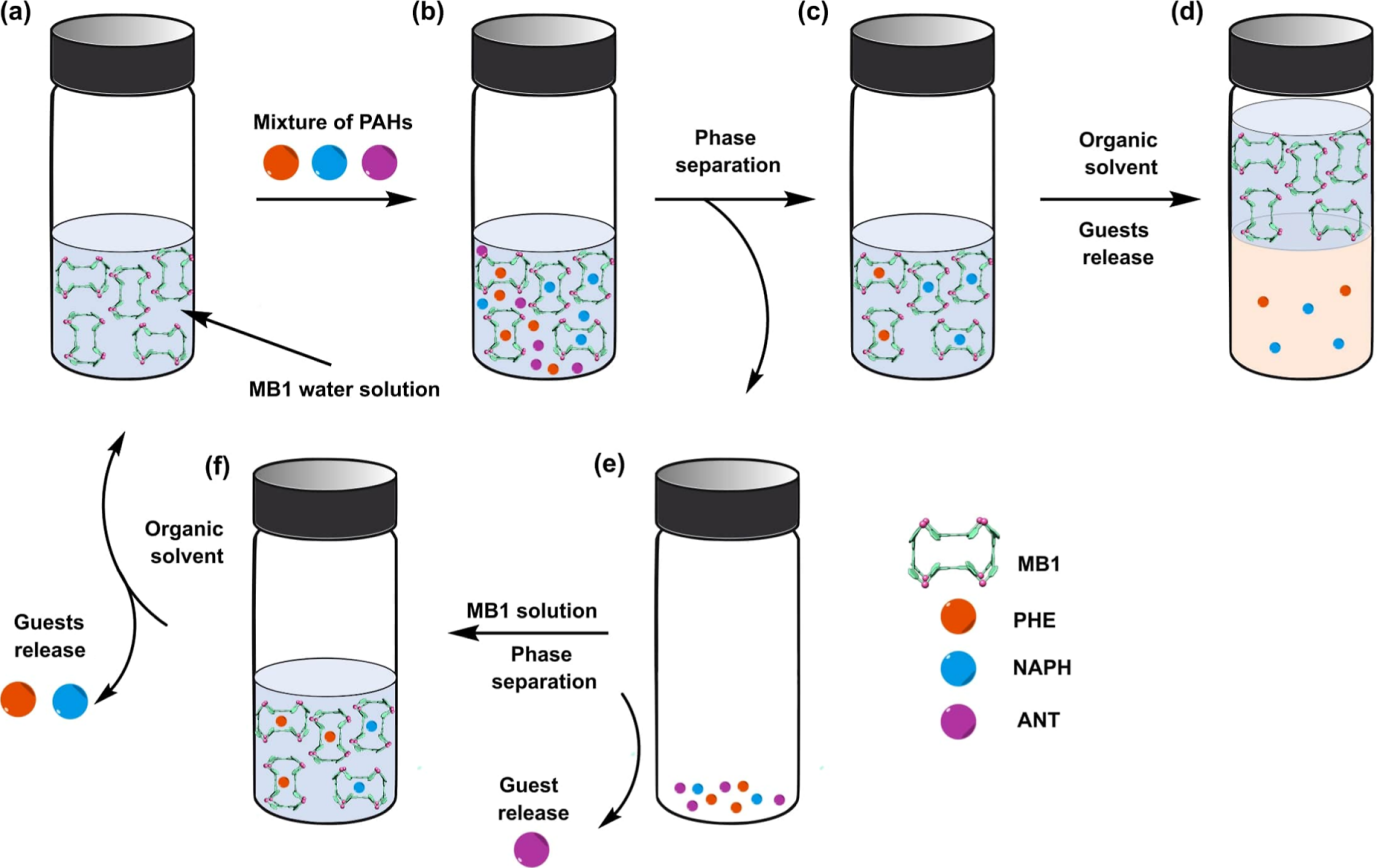
Stepwise separation of naphthalene (NAPH), phenanthrene (PHE), and anthracene (ANT) using **MB1** in water: (a) aqueous solution of **MB1**, (b) addition of a PAH mixture (NAPH, PHE, ANT) leading to selective encapsulation of NAPH and PHE, (c) phase separation by centrifugation to remove unbound ANT, (d) extraction with an organic solvent to release the encapsulated guests, (e) re-equilibration of the remaining PAH fraction with **MB1** solution, resulting in preferential encapsulation of PHE, (f) guest release and regeneration of **MB1** for reuse.

To quantify guest uptake during the initial encapsulation step, the aqueous phase was extracted with CDCl_3_ and analyzed by ^1^H NMR using 1,3,5-trimethoxybenzene as an internal standard. Unencapsulated guest molecules preferentially partition into the organic phase (CDCl_3_), whereas guests bound within the host cavity remain in the aqueous phase. Quantitative ^1^H NMR analysis of the CDCl_3_ extract therefore provides a direct measure of the relative amounts of unencapsulated guests and, by extension, their relative uptake during encapsulation.^51^ The molar ratio of naphthalene to phenanthrene in the CDCl_3_ extract was approximately 1.4:1, corresponding to 58% naphthalene and 42% phenanthrene.

The solid material isolated after the first centrifugation (containing PAHs that were not initially encapsulated) was resuspended in an aqueous solution of **MB1** and analyzed to determine its composition. The calculated molar ratio of naphthalene to phenanthrene in this second fraction was 1:2.9, corresponding to approximately 26% naphthalene and 74% phenanthrene (Figure S25). Anthracene remained exclusively in the solid phase throughout, consistent with its lack of encapsulation by **MB1** (Figure S24). Taken together, these results demonstrate that **MB1** is capable of selectively enriching and separating naphthalene and phenanthrene from mixed PAH samples in water. Importantly, **MB1** can be recovered through a simple centrifugation-extraction protocol: unbound PAHs were removed by centrifugation, followed by washing of the aqueous **MB1** phase with CDCl_3_ to release the encapsulated guests. The regenerated barrel was reused over three successive cycles without any loss of its selectivity (Figure S26).

## 3 Conclusion

In conclusion, we have demonstrated that local steric reinforcement in tetratopic N-donor ligands can decisively control the outcome of M_2*n*_L_*n*_-type metallosupramolecular self-assembly. Although M_2*n*_L_*n*_-type assemblies are typically obtained from tetratopic ligands combined with *cis*-blocked, square planar metal acceptors, the growing number of architectures that differ from the typical tetragonal prismatic topology highlights that the factors governing their formation remain incompletely understood. Recent studies have identified several key parameters, including donor ligand geometry, size, and the nature of the metal acceptor, as crucial determinants of the final architecture.^29,66,68^ In this work, we show that ligands of comparable overall shape and width can lead to entirely different assemblies. The tetrapyridylbiphenyl ligand (**L**), equipped with two sterically demanding ethoxy substituents, restricts rotation around the C_phenyl_–C_pyridyl_ bond and directs formation of a single, well-defined tetragonal prismatic barrel (**MB1**). In contrast, the unsubstituted analogue undergoes unrestricted conformational sampling and forms a mixture of assemblies under identical conditions. These findings establish local steric reinforcement serves as a decisive element in achieving conformational control and exclusive single-cage formation. The resulting water-soluble barrel **MB1** exhibits size-selective encapsulation of smaller PAHs. Its narrow, elongated hydrophobic cavity encapsulates naphthalene and phenanthrene, consistent with superior size complementarity and enhanced hydrophobic stabilization within the confined cavity. Consequently, **MB1** functions as a “separation flask”, enabling the sequential identification, enrichment, and separation of naphthalene, phenanthrene, and anthracene directly in aqueous solution through a simple phase separation.

## 4 Experimental Section

### 4.1 Synthesis of Ligand (L)

The ligand **L** was synthesized with a modification of the previously described procedure,^67^ following the same synthetic route also used to obtain subcomponents **2** and **3**. A solution of K_2_CO_3_ (5.2 g, 37.7 mmol) in H_2_O (8 mL) and THF (30 mL) was placed in a Schlenk flask and degassed by applying vacuum, followed by three cycles of argon flow. To the degassed solution, compound **3** (1.2 g, 2.6 mmol), 3-pyridinylboronic acid (2.0 g, 16.3 mmol) and Pd­(PPh_3_)_4_ (160 mg, 0.13 mmol) were added under an argon flow. The reaction mixture was heated at 70 °C for 72 h. After cooling to room temperature, the mixture was combined with 50 mL of H_2_O and extracted with 3 × 150 mL portions of CHCl_3_. The organic layers were collected and dried over anhydrous Na_2_SO_4_. The organic solvents were then removed using a rotary evaporator. The obtained off-white solid was washed with cold MeOH to yield the ligand L as a white solid (1.1 g, 92% yield). ^1^H NMR (300 MHz, CDCl_3_): δ (ppm) = 8.92 (d, 4H), 8.63 (d, 4H), 8.03 (m, 4H), 7.63 (s, 4H), 7.40 (m, 4H), 3.33 (m, 4H), 0.84 (t, 6H). ^13^C NMR (125 MHz, CDCl_3_): δ (ppm) = 154.15, 150.16, 148.93, 136.88, 136.79, 134.11, 133.61, 129.44, 123.23, 69.68, 15.36. HRMS-ESI: calcd 551.2441; found, 551.2421 (*m*/*z*).

### 4.2 Synthesis of MB1

**L** (10 mg, 0.018 mmol) was added to a 0.5 mL H_2_O/CD_3_CN (4:1) solution containing M (12.6 mg, 0.036 mmol) followed by constant stirring at 60 °C for 24 h. The resulting clear, pale yellow solution was subsequently treated with an excess of THF, leading to the isolation of a white solid corresponding to the **MB1**. Yield: 20.5 mg (22%). ^1^H NMR (D_2_O/CD_3_CN 4:1): δ (ppm): 9.83 (s, 8H), 9.32 (s, 8H), 9.14 (s, 8H), 8.87 (d, 8H), 8.47 (d, 8H), 8.19 (d, 8H), 8.01 (m, 8H), 7.87 (s, 8H), 7.80 (s, 8H), 7.72 (m, 8H), 3.75 (d, 8H), 3.09–3.04 (m, 32H), 2.56 (d, 8H), 0.95 (t, 12H), −0.66 (t, 12H). HRMS-ESI (*m*/*z*): [**MB1**•10PF_6_^–^]^6+^ calcd 830.3969; found, 830.3969; [**MB1**•11PF_6_^–^]^5+^ calcd 1026.4764; found, 1026.4695; [**MB1**•12PF_6_^–^]^4+^ calcd 1318. 0774; found, 1318.0748; and [**MB1**•13PF_6_^–^]^3+^ calcd 1806.7652; found, 1806.7626 *m*/*z* [M + H]^+^.

### 4.3 X-ray Crystallography

Crystal Data for C_476_H_204_N_38_O_19_Pd_8_ (M = 7609.96 g/mol): triclinic, space group *P*1 (no. 2), *a* = 16.075(3) Å, *b* = 19.113(4) Å, *c* = 24.242(5) Å, α = 83.49(3)°, β = 88.57(3)°, γ = 67.54(3)°, *V* = 6837(3) Å^3^, *Z* = 1, *T* = 100(2) K, μ­(synchrotron) = 0.612 mm^–1^, *D*_calcd_ = 1.848 g/cm^3^, 28,332 reflections measured (1.692° ≤ 2Θ ≤ 34.454°), 7968 unique (*R*_int_ = 0.1456, *R*_sigma_ = 0.1248) which were used in all calculations. The final *R*_1_ was 0.1339 (I > 2σ­(I)) and *wR*_2_ was 0.4135 (all data).

### 4.4 General Procedure for Host–Guest Complexes

In a 5 mL glass vial, **MB1** (10 mg, 0.0022 mmol) and an excess amount of the guest molecules (ca. 5 equiv) were combined with a D_2_O/CD_3_CN (4:1) solution (0.5 mL) and stirred for 24 h at 60 °C. The unencapsulated excess guest was subsequently removed by filtration. The resulting clear solution, containing the guest⊂**MB1** inclusion complex, was examined by NMR spectroscopy.

## Supplementary Material



## Notes

Additional experimental details, materials and methods, XRD data, and supplementary figures (PDF). (PDF)
