# Supplementary material for: Ligand Side-Chains Control the Formation of an M8L4 Molecular Barrel: Unveiling Selective Encapsulation and Sequential Separation Properties
Source: Inorg Chem. 2026 Mar 19;65(12):6913–22. doi: 10.1021/acs.inorgchem.6c00394 (PMC13299013; doi:10.1021/acs.inorgchem.6c00394)
Supplement: Supplementary file 1 [file ic6c00394_si_001.pdf]

Supporting Information

Ligand Side-Chains Control the Formation of an M_8L_4 Molecular Barrel: Unveiling Selective Encapsulation and Sequential Separation Properties

Agnieszka Bajer,[1,2] Venkateswarulu Mangili,[1] Atiqur Rahman,[3] Jack K. Clegg,[3] Artur R. Stefankiewicz[1,2,]***

[1] Center for Advanced Technologies, Adam Mickiewicz University, Uniwersytetu Poznańskiego 10, 61-614 Poznań, Poland

[2] Faculty of Chemistry, Adam Mickiewicz University, Uniwersytetu Poznańskiego 8, Poznań, 61-614 Poland

[3] School of Chemistry and Molecular Biosciences, The University of Queensland, St. Lucia, Queensland 4072, Australia.

*Correspondence: venkateswarulu.mangili@amu.edu.pl

**Correspondence: ars@amu.edu.pl

Contents:

1. Materials and methods 2
2. Spectral characterization of **L** 3
3. Spectral characterization of **MB1** 4
4. X-Ray crystallographic study of **MB1** 9
5. General procedure for host-guest complexes 10
6. Guest binding studies 15
7. Selective host-guest chemistry 20
8. References 23

1. Materials and methods

General chemicals and the solvents were purchased from commercially available suppliers (Fluorochem and Merck) and were used without further purification. NMR solvents were provided by Deutero GmbH and used as received. NMR spectra were recorded at 298 K on Bruker Fourier 300 MHz, 400 MHz and Bruker Avance IIIHD 600 MHz spectrometers, with solvent residual peaks serving as internal references: D_2O (δ = 4.79 ppm), $CDCl_3$ (δ = 7.26 ppm), CD_3CN (δ = 1.94 ppm) and DMSO-d_6 (δ = 2.50 ppm). ^{13}C NMR spectra were recorded using the same instruments at 75 MHz and all the chemical shifts (δ) were reported in ppm relative to external $CDCl_3$ (77.8-77.2 ppm), CD_3CN (1.32, 118.26 ppm) and DMSO-d_6 (39.52 ppm). All NMR data were processed using Mestrelab Research MNova software.

ESI-MS spectra were acquired on a Bruker HD Impact spectrometer in positive ion mode. Typical acquisition parameters were as follows: capillary voltage 3.6 kV, nebulizer pressure 0.5 bar, drying gas flow 4.0 L·min^{-1}, drying gas temperature 200 ˚C, and an m/z range of 300-2500. Theoretical MS spectra were predicted using Mestrelab Research MNova software.

Photoluminescence spectra were recorded on a Jasco FP-8300 spectrofluorometer using quartz cuvettes (1 cm path length). Measurements were performed in emission mode with excitation and emission slit widths set to 2.5 nm. Spectra were collected using a xenon lamp as the excitation source with a response time of 0.5 s and high detector sensitivity. The excitation wavelength was set to 275 nm (for NAPH) and 295 nm (for PHE), and emission was recorded in the corresponding wavelength ranges. Spectral correction was applied in all measurements.

2. Spectral characterization of **L**

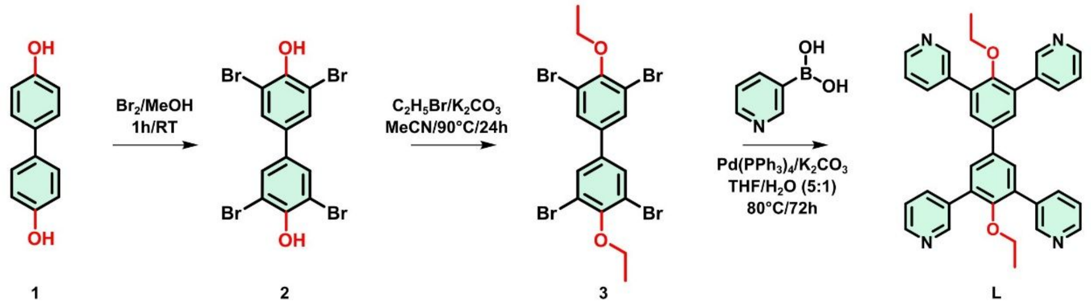

Scheme S1: Synthetic route for the preparation of the ligand **L**.

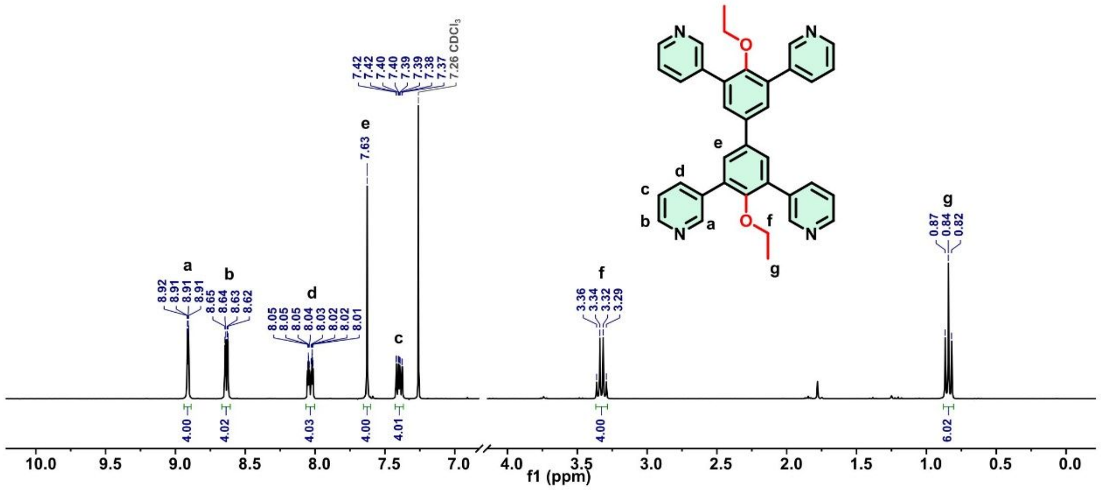

Figure S1. ^{1}H NMR (300 MHz, $CDCl_3$) of **L**.

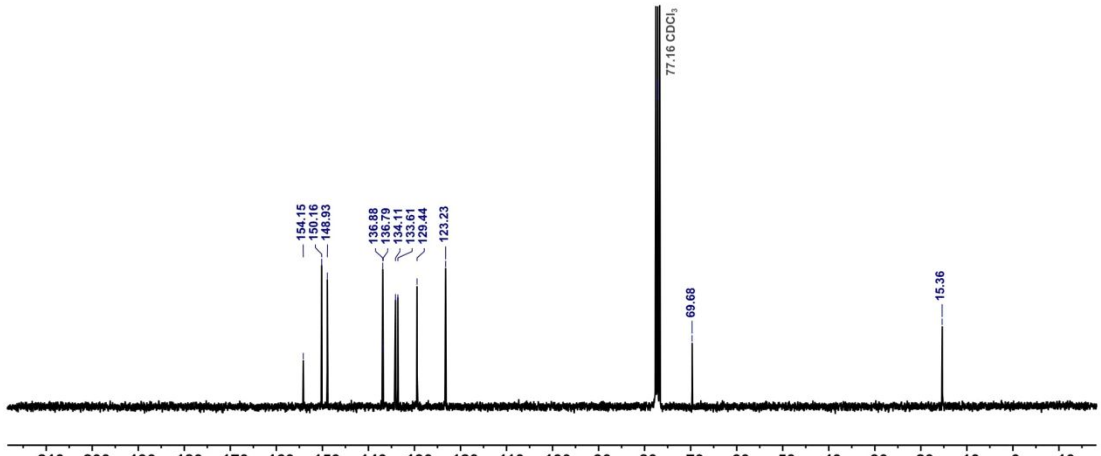

Figure S2. ^{13}C NMR (75 MHz, $CDCl_3$) of **L**.

Ligand samples were analyzed directly from chloroform solutions without further purification.

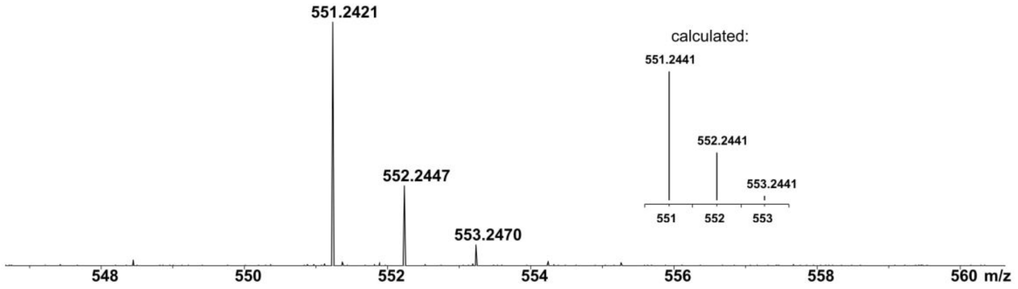

Figure S3: ESI-MS of **L**.

3. Spectral characterization of **MB1**

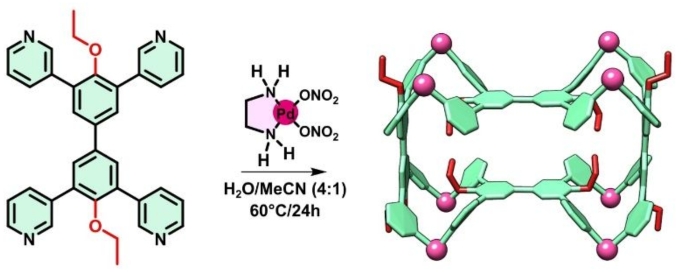

Scheme S2: Synthetic route for the preparation of the molecular barrel **MB1**.

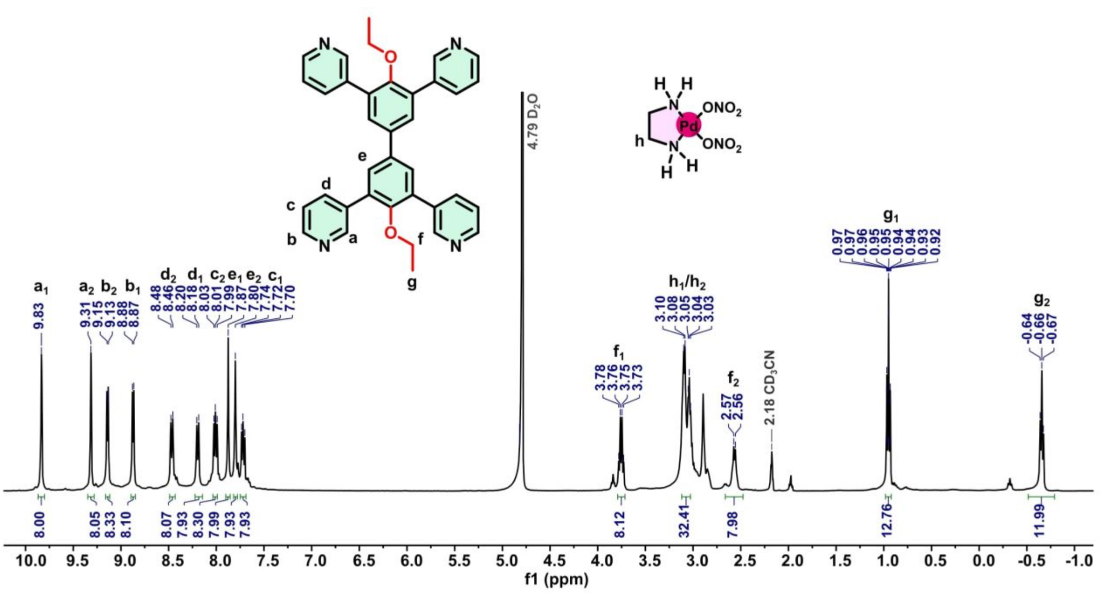

Figure S4. ^{1}H NMR (300 MHz, D_2O:CD_3CN (4:1 v/v)) of **MB1**.

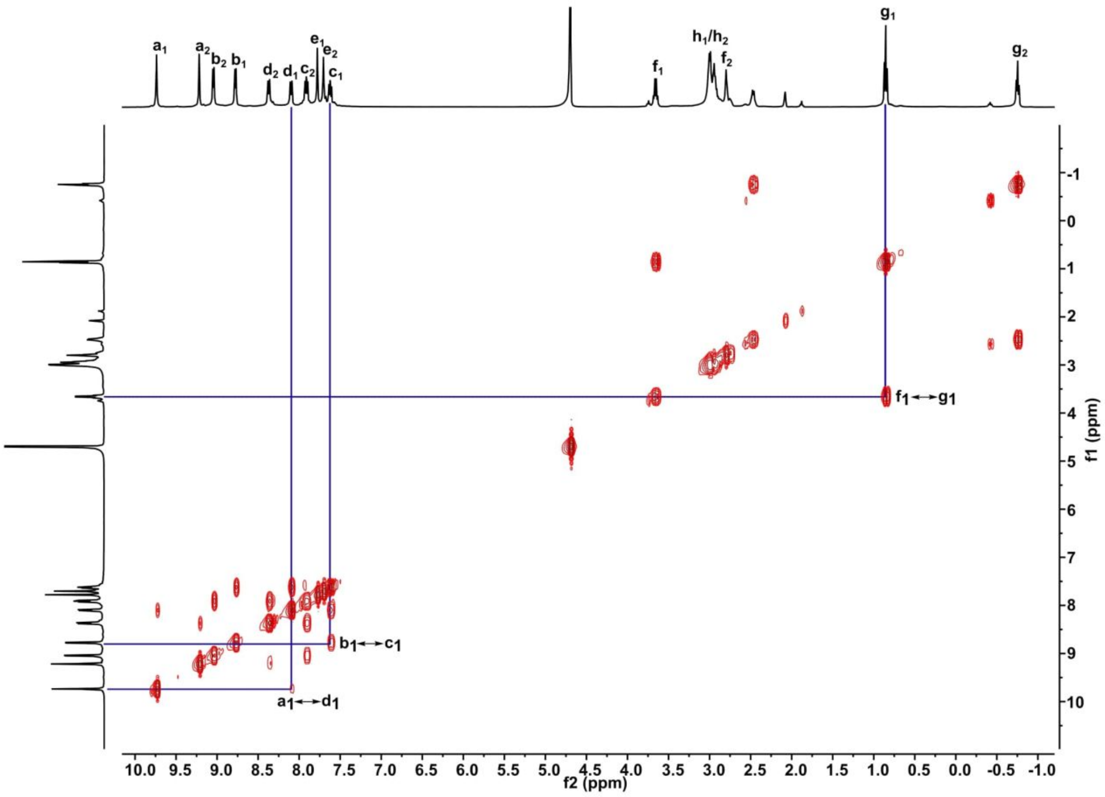

Figure S5: ^{1}H-^{1}H COSY NMR (300 MHz, D_2O:CD_3CN (4:1 v/v)) **MB1**.

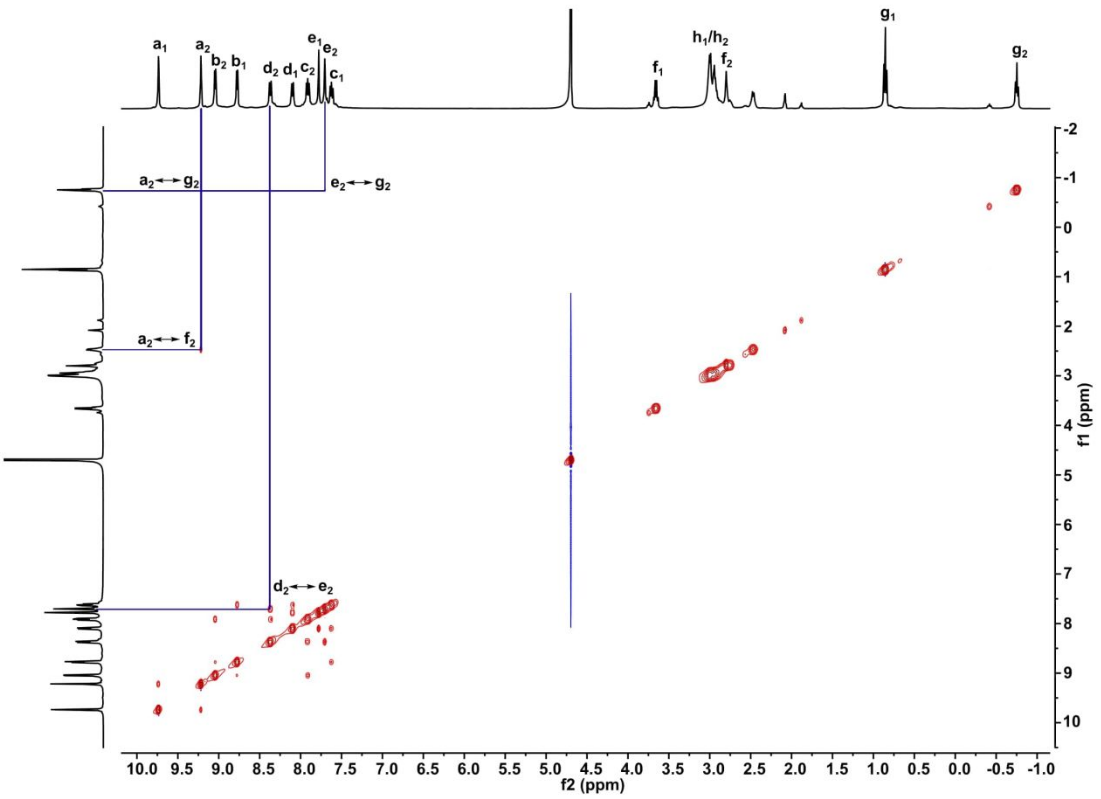

Figure S6: ^{1}H-^{1}H NOESY NMR (300 MHz, D_2O:CD_3CN (4:1 v/v)) **MB1**.

DOSY NMR experiments revealed a single diffusing species for **MB1** with a diffusion coefficient of log$D$ = -9.55. Hydrodynamic radii were estimated from the diffusion coefficients using the Stokes-Einstein equation, assuming a spherical approximation. Based on the measured diffusion coefficient at 298 K, a hydrodynamic radius of approximately 3.9 Å (hydrodynamic diameter ca. 7.8 Å) was obtained. This value is comparable to the transverse dimension of the internal cavity (ca. 8.6 Å) observed in the solid-state structure, supporting the retention of the cage architecture in solution.

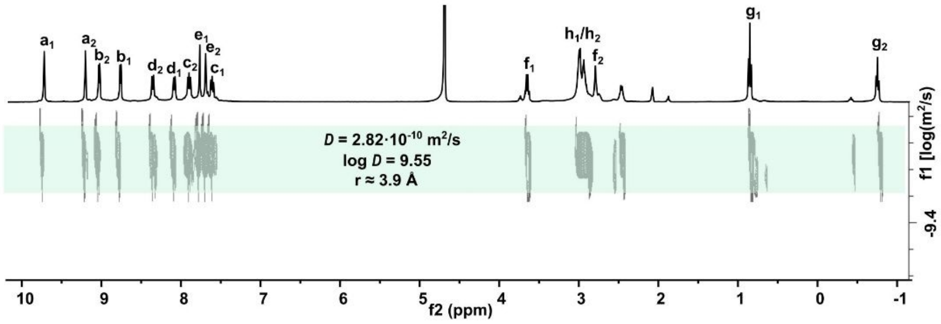

Figure S7: ^{1}H DOSY NMR (600 MHz, D_2O:CD_3CN (4:1 v/v)) **MB1**.

For ESI-MS analysis, the PF_6^- salt of **MB1** was prepared by adding an excess of KPF_6 to an aqueous solution of the corresponding nitrate salt to induce anion exchange. The resulting precipitate was collected by filtration, and thoroughly washed with water to remove residual inorganic salts, and subsequently redissolved in acetonitrile. The solution was further diluted to micromolar concentrations immediately before ESI-MS measurements.

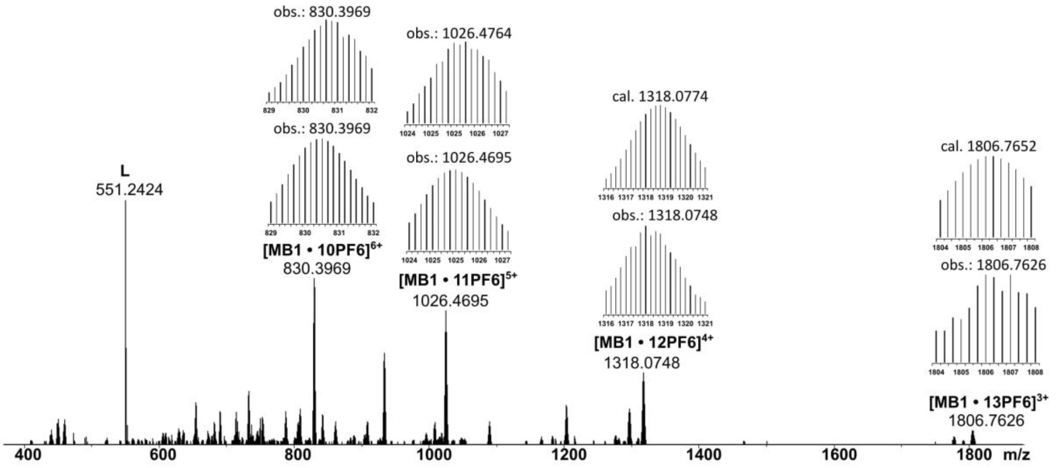

Figure S8: ESI-MS spectrum of the PF_6^- analogue of **MB1** in CH_3CN.

Variable-Temperature (VT) NMR Studies

Samples of **MB1** were prepared at a concentration of ca. 2.4 mM in D_2O and in a D_2O/CD_3CN (4:1 v/v) solvent mixture. ^{1}H NMR spectra were recorded on a 400 MHz spectrometer over the temperature range of 10-60°C. Chemical shifts were referenced to residual solvent signals.

Throughout the investigated temperature range, the resonances corresponding to the ethoxy substituents remained as a single set of signals, showing gradual downfield shifts with increasing temperature and no evidence of peak splitting or coalescence.

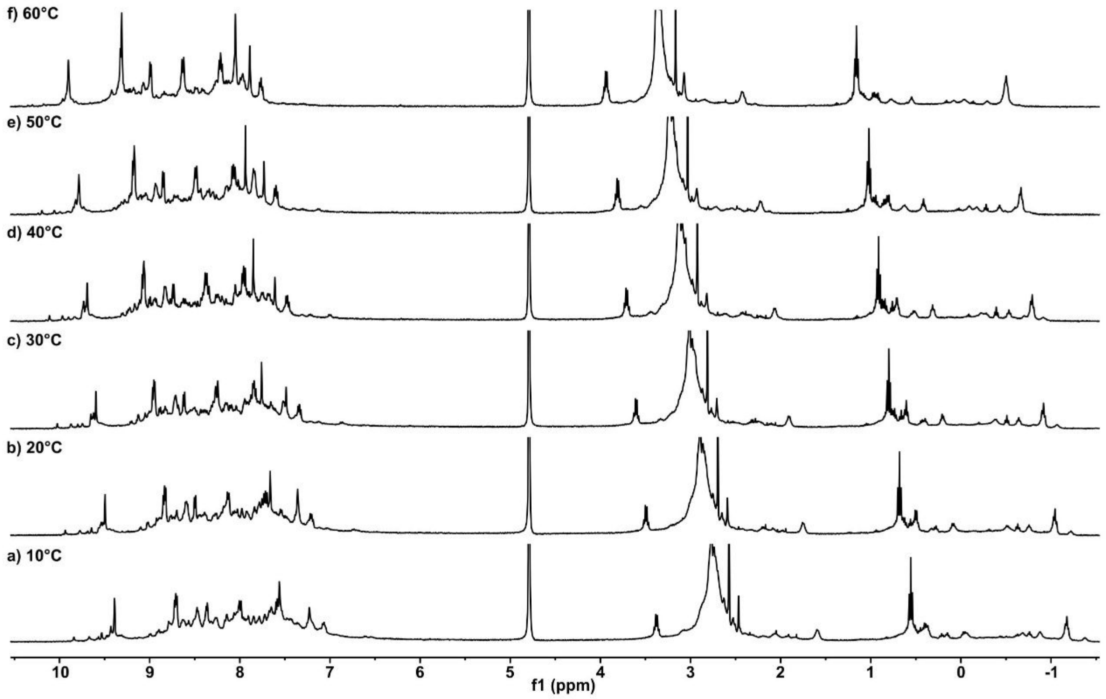

Figure S9: Variable temperature ^{1}H NMR (400 MHz, D_2O) of **MB1**.

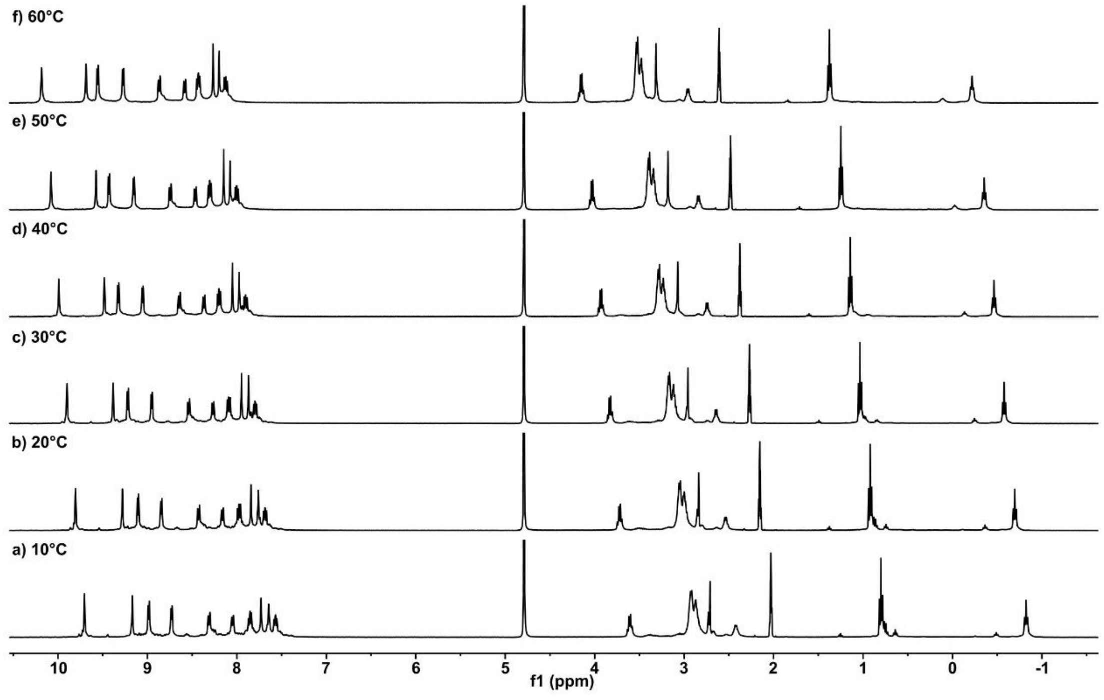

Figure S10: Variable temperature ^{1}H NMR (400 MHz, D_2O:CD_3CN (4:1 v/v)) of **MB1**.

4. X-Ray crystallographic study of **MB1**

Single crystal X-ray data were collected using Silicon Double Crystal monochromated synchrotron radiation at 100(2) K at the MX1 beamline of the Australian synchrotron.[1, 2] Data integration and reduction were performed using XDS.[3] The structures were solved by intrinsic phasing using ShelXT[4] and refined by the full-matrix least-squares method using SHELXL through the Olex2 GUI.[5] Non-hydrogen atoms were refined anisotropically, carbon-bound hydrogen atoms were included in idealized positions and refined using a riding model. The crystals yielded less than ideal diffraction quality despite the use of synchrotron radiation and no reflections at better than 1.0 Å resolution were observed. The connectivity of the structure, however, is unambiguous. In part the low resolution of the dataset likely arises from large areas of smeared electron density that were present due to disordered solvent molecules and/or anions which were treated with a solvent mask.[6]

Crystallographic data and refinement parameters are given below (CCDC # 2522966). Two level A and 11 level B CheckCIF alerts remain which are due to the lower than ideal resolution of the dataset as discussed above.

5. General procedure for host-guest complexes

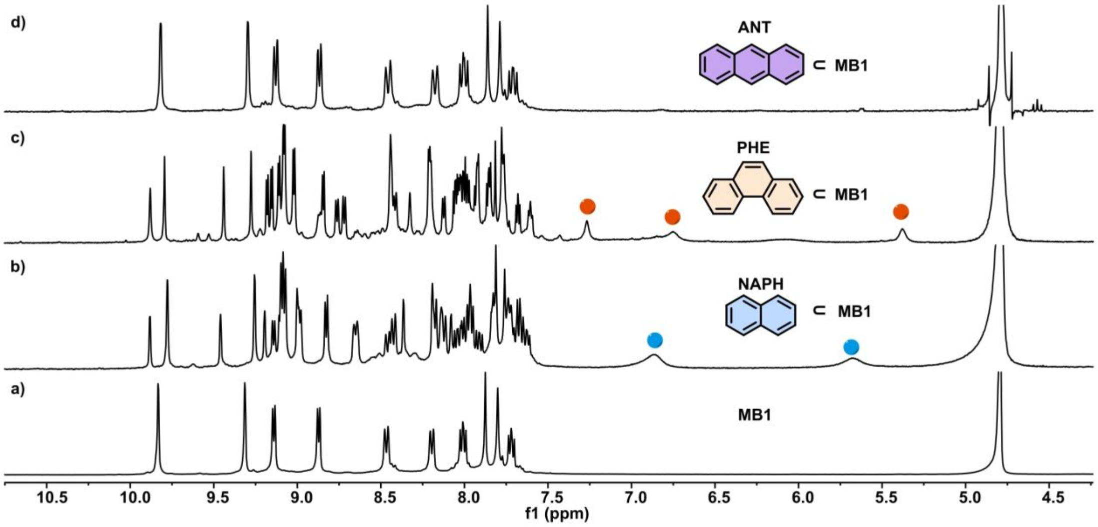

Figure S11. ^{1}H NMR (300 MHz, D_2O:CD_3CN (4:1 v/v)) stack plot of a) **MB1**, b) NAPH□**MB1** (NAPH: Naphthene), c) PHE□**MB1** (PHE: Phenanthrene), d) ANT□**MB1** (ANT: Anthracene) showing the change in NMR by guest encapsulation.

The DOSY NMR spectrum of PHE□**MB1** reveals a single diffusing species with log D = -9.63, corresponding to a calculated hydrodynamic radius of ~ 4.7 Å.

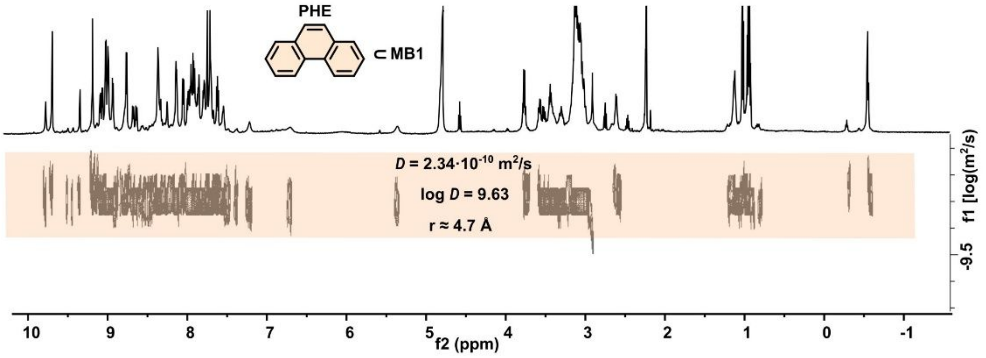

Figure S12. ^{1}H DOSY NMR (600 MHz, D_2O:CD_3CN (4:1 v/v)) of PHE□**MB1** (PHE: Phenanthrene).

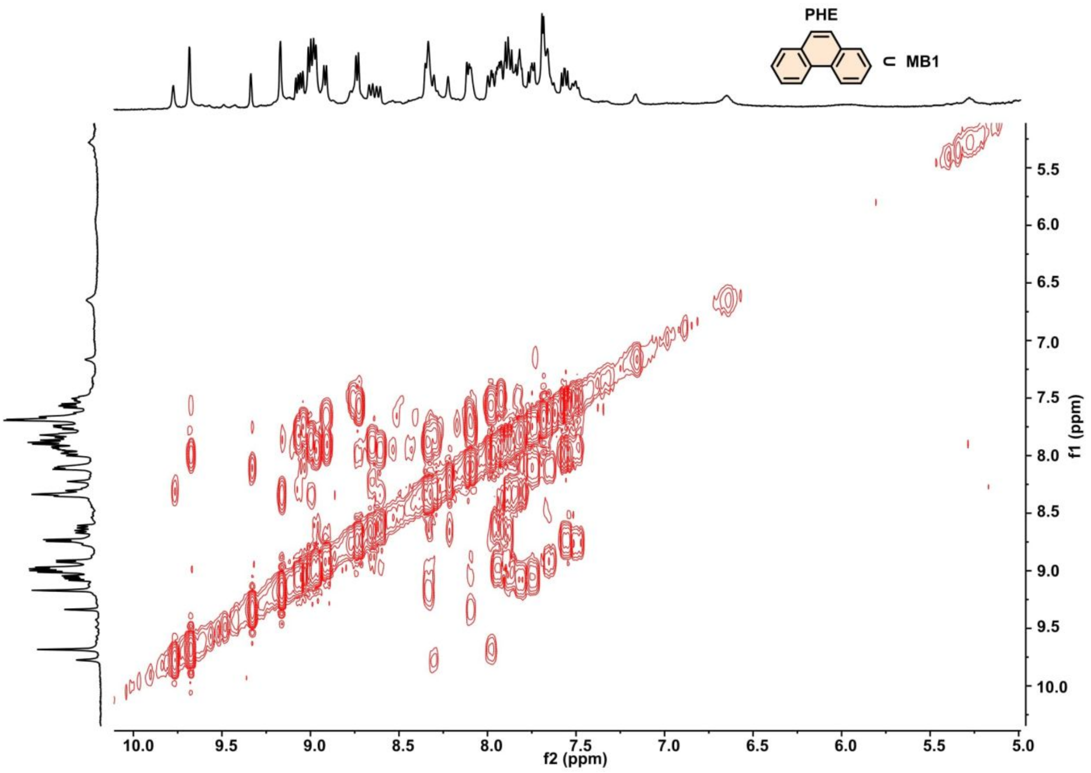

Figure S13: ^{1}H-^{1}H COSY NMR (300 MHz, D_2O:CD_3CN (4:1 v/v)) of PHE⊂**MB1** (PHE: Phenanthrene).

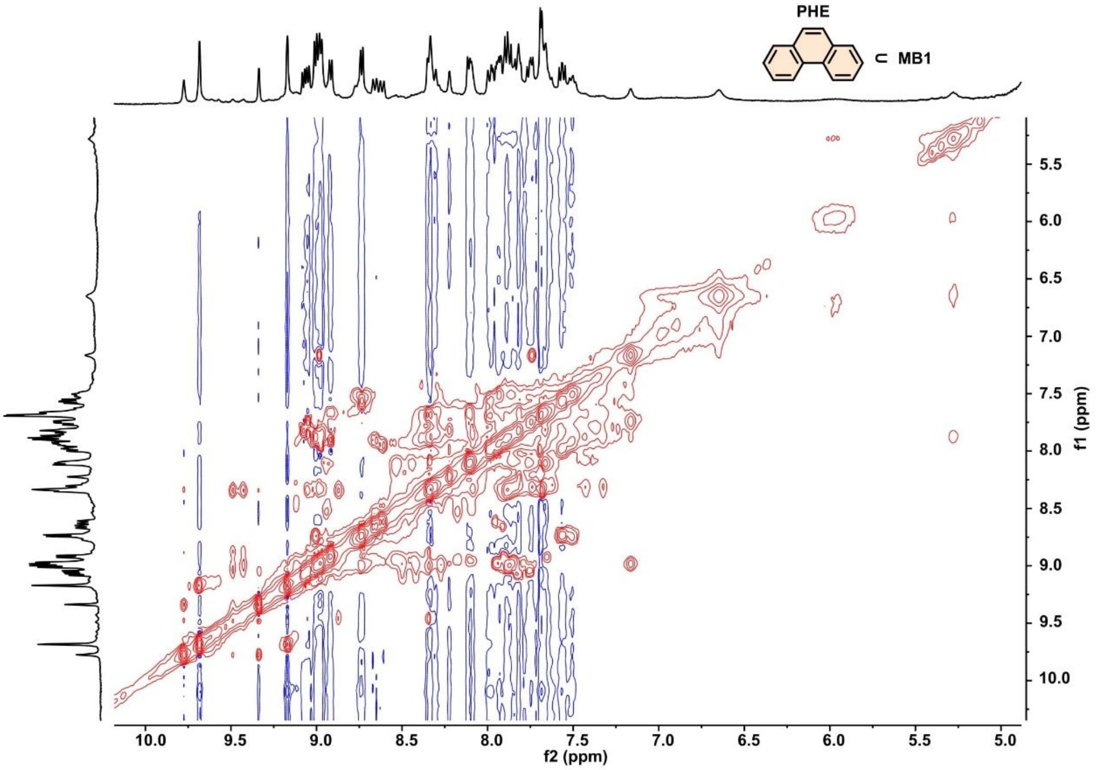

Figure S14: ^{1}H-^{1}H NOESY NMR (300 MHz, D_2O:CD_3CN (4:1 v/v)) of PHE⊂**MB1** (PHE: Phenanthrene).

The DOSY NMR spectrum of NAPH⊂**MB1** reveals a single diffusing species with log D = -9.65, corresponding to a calculated hydrodynamic radius of ~ 4.9 Å.

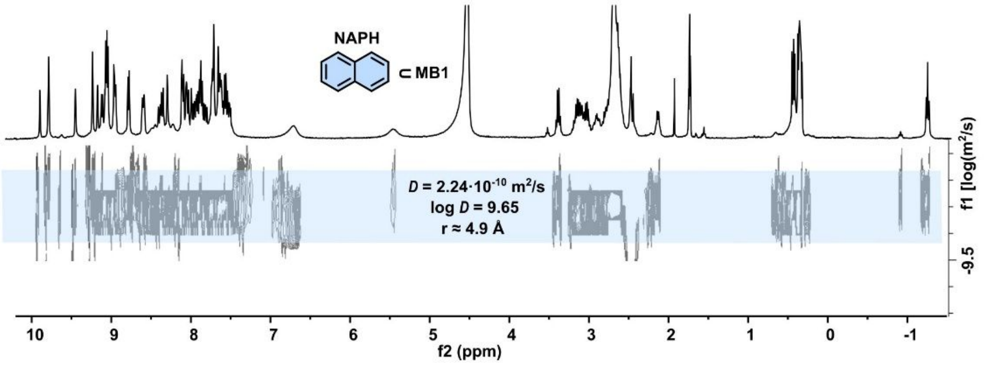

Figure S15. ^{1}H DOSY NMR (600 MHz, D_2O:CD_3CN (4:1 v/v)) of NAPH⊂**MB1** (NAPH: Naphthalene).

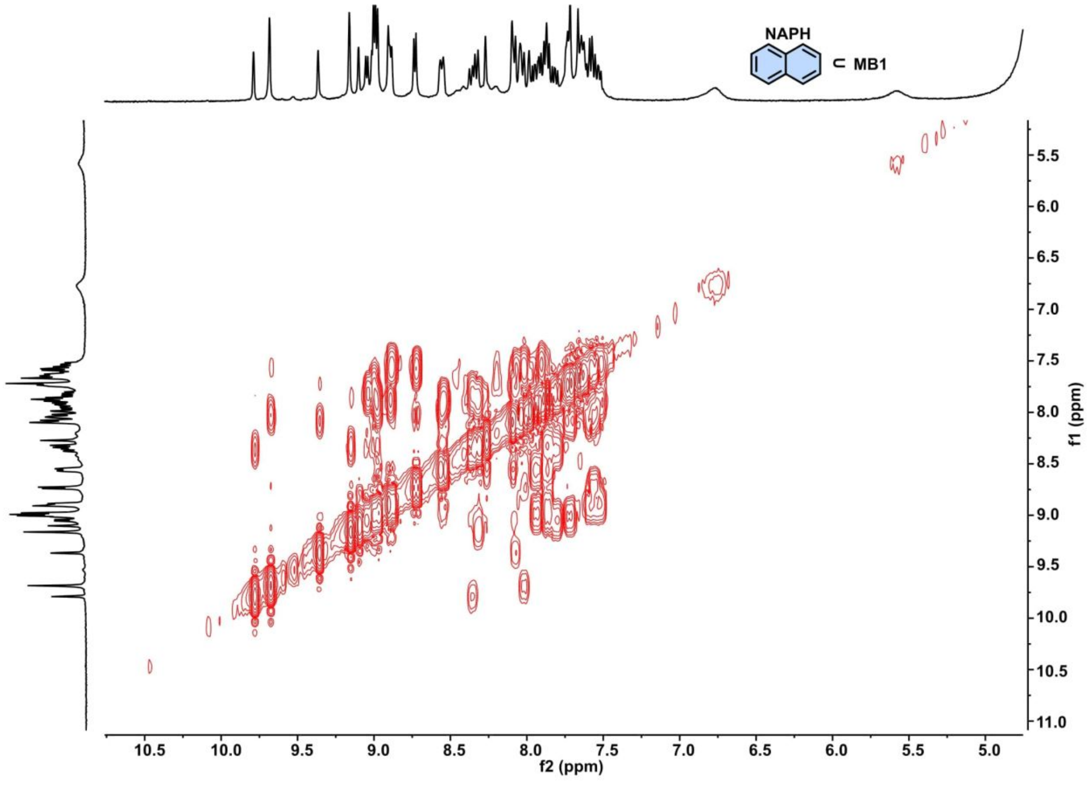

Figure S16: ^{1}H-^{1}H COSY NMR (300 MHz, D_2O:CD_3CN (4:1 v/v)) of NAPH⊂**MB1** (NAPH: Naphthalene).

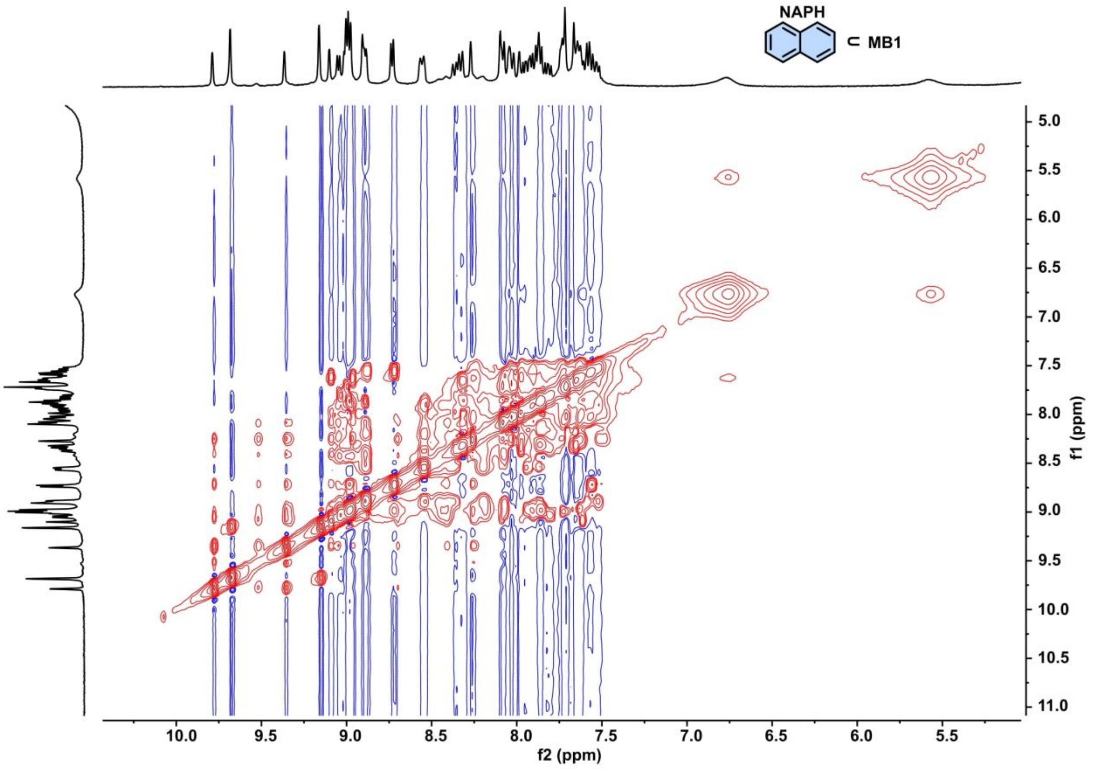

Figure S17: ^{1}H-^{1}H NOESY NMR (300 MHz, D_2O:CD_3CN (4:1 v/v)) of NAPH□**MB1** (NAPH: Naphthalene) in D_2O/CD_3CN (4:1, v/v).

Photoluminescence Measurements

Fluorescence spectra were recorded for **MB1**, NAPH, PHE, NAPH⊂**MB1**, and PHE⊂**MB1** in aqueous medium under identical conditions. Samples of the host-guest complexes were prepared by stirring **MB1** with an 1eq of the corresponding guest for 24 h at room temperature, followed by removal of undissolved guest by centrifugation. The final concentration of all samples was adjusted to 8 μM.

Emission spectra were collected on spectrofluorometer at room temperature using quartz cuvettes (1 cm path length). For NAPH and NAPH⊂MB1, spectra were recorded with excitation at 275 nm over the emission range of 300-400 nm. For PHE and PHE⊂**MB1**, excitation was set to 295 nm and emission was monitored over 335-435 nm. Spectra were corrected for background and solvent contributions.

Under these conditions, the free guests exhibited very weak fluorescence in aqueous medium due to their low solubility, whereas pronounced emission was observed for the corresponding host-guest complexes.

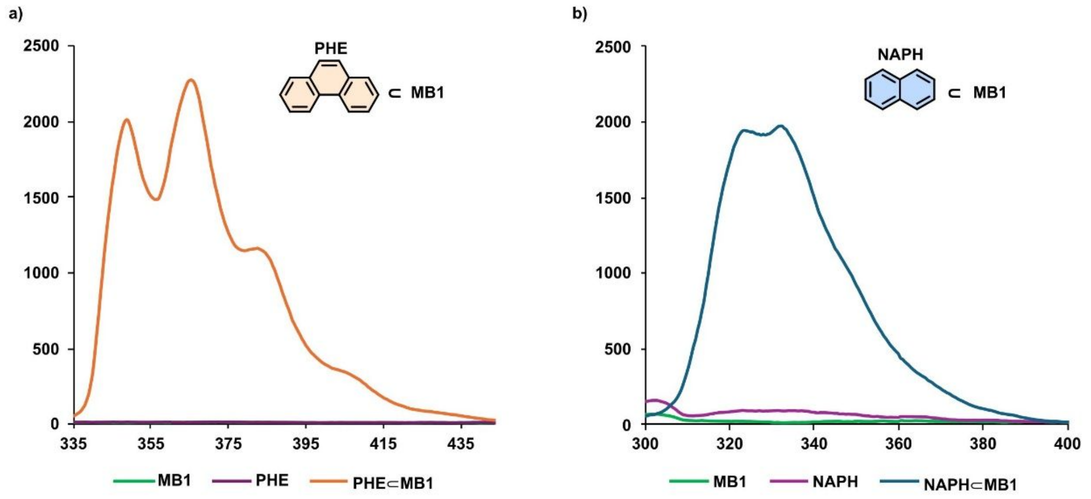

Figure S18: Fluorescence emission spectra of a) **MB1**, PHE and PHE□**MB1**, b) **MB1**, NAPH and NAPH□**MB1** in H_2O.

6. Guest binding studies

The host-guest interactions between **MB1** and the two aromatic guests, phenanthrene (PHE) and naphthalene (NAPH), were investigated using ^1H NMR titration in D_2O. Preliminary titrations performed in mixed D_2O/CD_3CN-d_3 solvent exhibited unstable and irreproducible chemical shift changes due to partial solvation of the guests; therefore, all final titrations were carried out in pure D_2O, where the observed chemical shift responses were consistent and well-defined.

A stock solution of **MB1** was titrated by stepwise addition of aliquots of guest solution (0.4 equivalents per addition). After each addition, ^1H NMR spectra were recorded at 25°C. For both guests, the system exhibited slow exchange on the NMR timescale, allowing separate observation of the signals corresponding to the free host (H) and the host-guest complex (HG).

The progress of binding was monitored using the aromatic proton H_2 of **MB1**, whose chemical shift changed systematically upon guest binding. The fraction of bound host sites, θ, was calculated from the observed chemical shift changes according to:

$$\theta = \frac{\delta - \delta_{\text{free}}}{\delta_{\text{bound}} - \delta_{\text{free}}}$$

where $\delta_{\text{free}}$and δ_{bound} are the chemical shifts of the free host and the fully bound host, respectively.

The apparent association constant (K_a) and the Hill coefficient (n), describing the degree of cooperative binding, were determined using the Hill equation[7]:

$\log\left(\frac{\theta}{1-\theta}\right) = n\log[G] + \log K_a$where $[G]$ is the total guest concentration. The values of K_a and n were obtained from linear regression of the Hill plot ($\log(\theta/(1-\theta))$ vs. $\log[G]$). This analysis yielded

the apparent host-guest association constants and cooperativity parameters for the **MB1**·PHE and **MB1**·NAPH complexes.

Calculation of Standard Gibbs Free Energy (ΔG°) for Host–Guest Complexation

The thermodynamic stability of each host-guest complex was quantified by converting the association constant $K_a$obtained from Hill-model fitting into the corresponding standard Gibbs free energy of binding (ΔG°). All calculations were performed at 298 K.

The standard Gibbs free energy of binding is related to the equilibrium constant according to the fundamental thermodynamic equation:

$$\Delta G^{\circ} = -RT\ln K_a$$

where:

- R – universal gas constant R = 8.314 J/mol·K
- T – temperature in Kelvin T = 298 K
- K_a – association constant derived from Hill analysis or BindFit.

A larger K_a corresponds to more negative ΔG°, meaning stronger binding.

6.1. Binding of phenanthrene

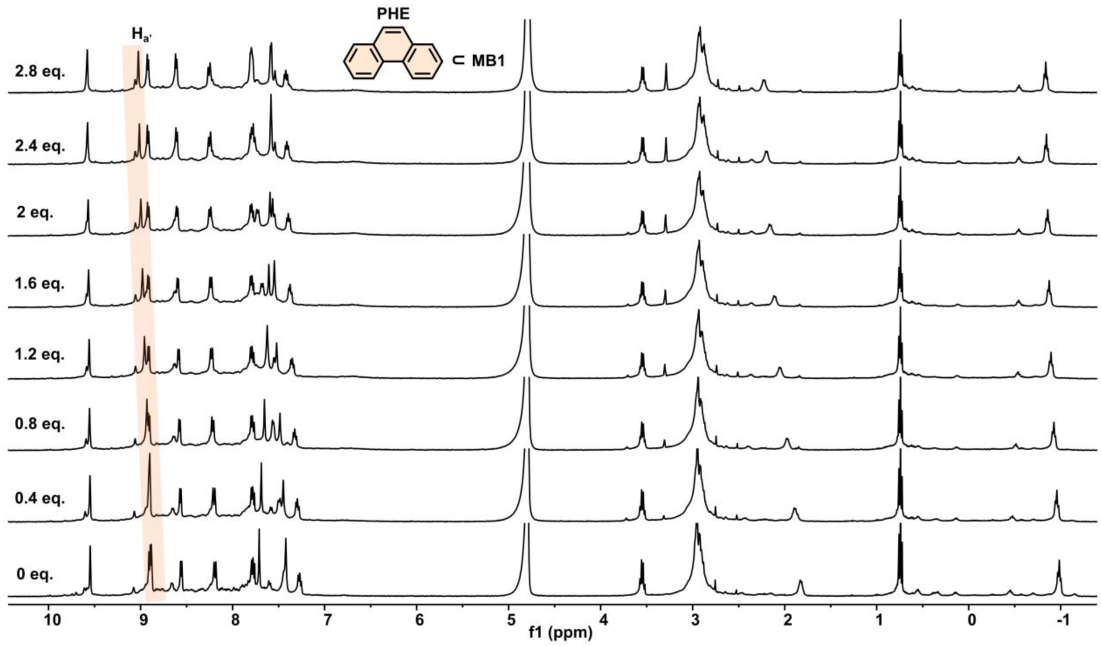

Figure S19: ^{1}H NMR (300 MHz, D_2O) titration of **MB1** (c = 4.481 mM) upon addition of 0 - 2.8 eq. of PHE (Phenanthrene). $H_{a'}$ proton signals of **MB1** are highlighted in orange.

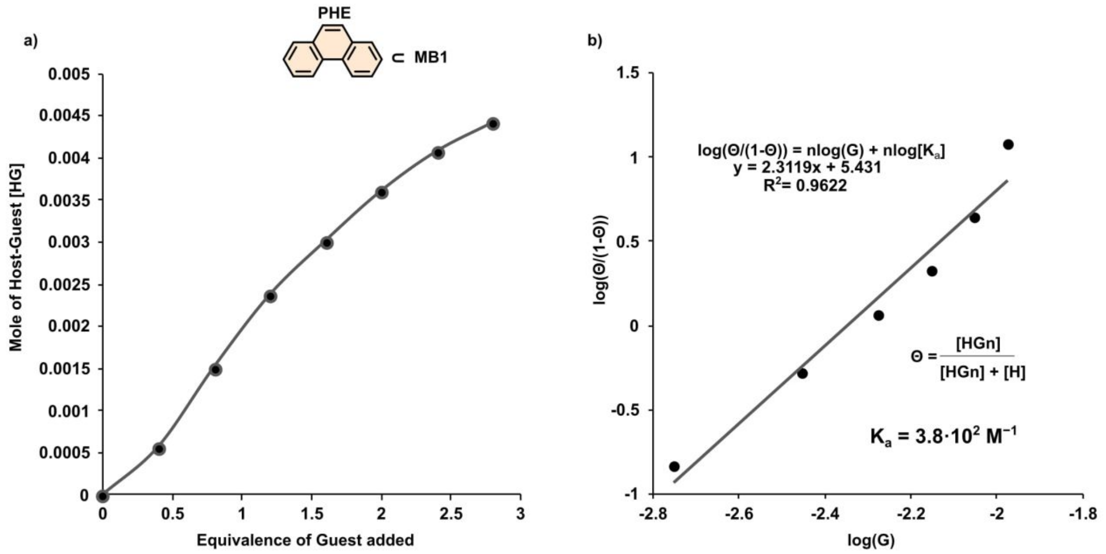

Figure S20: Titration curve of **MB1** with PHE (Phenanthrene) a) plot of Mole of Host-Guest [HG] formed vs Equivalents of Guest added b) plot of $log(\theta/(1-\theta))$ vs log[G].

$$\Delta G^{\circ} = -RTln(3.8 \cdot 10^2)$$

$$\Delta G^{\circ} = -14.73 \text{ kJ/mol}$$

6.2. Binding of naphthalene

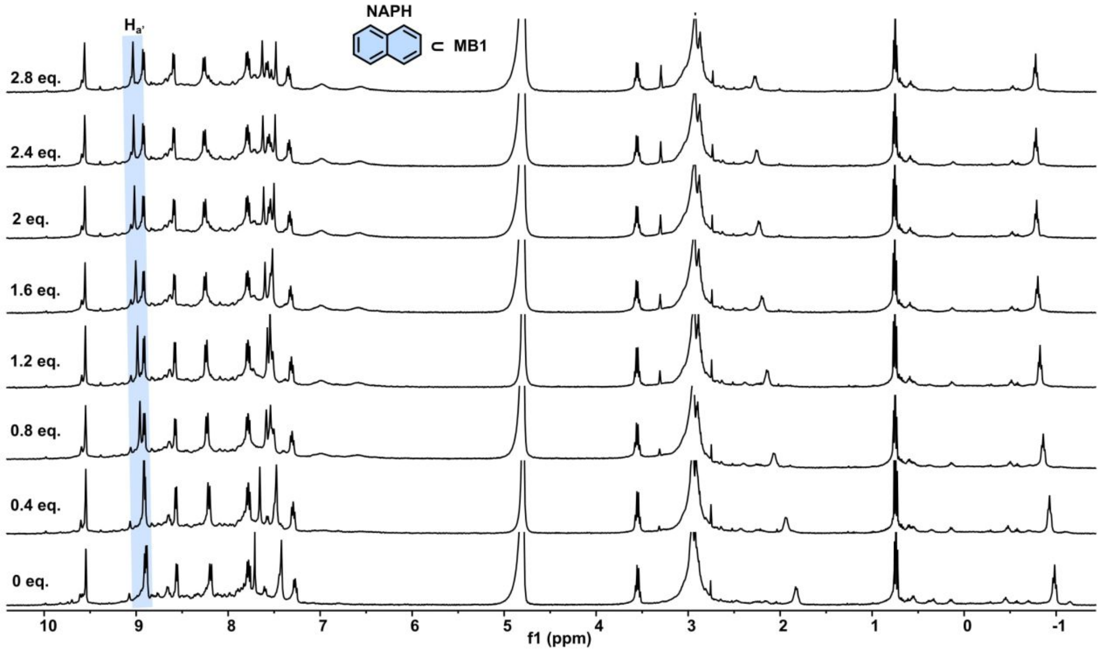

Figure S21: ^{1}H NMR (300 MHz, D_2O) titration of **MB1** (c = 4.481 mM) upon addition of 0 - 2.8 eq. of NAPH (Naphthalene). $H_{a'}$ proton signal of **MB1** is highlighted in blue.

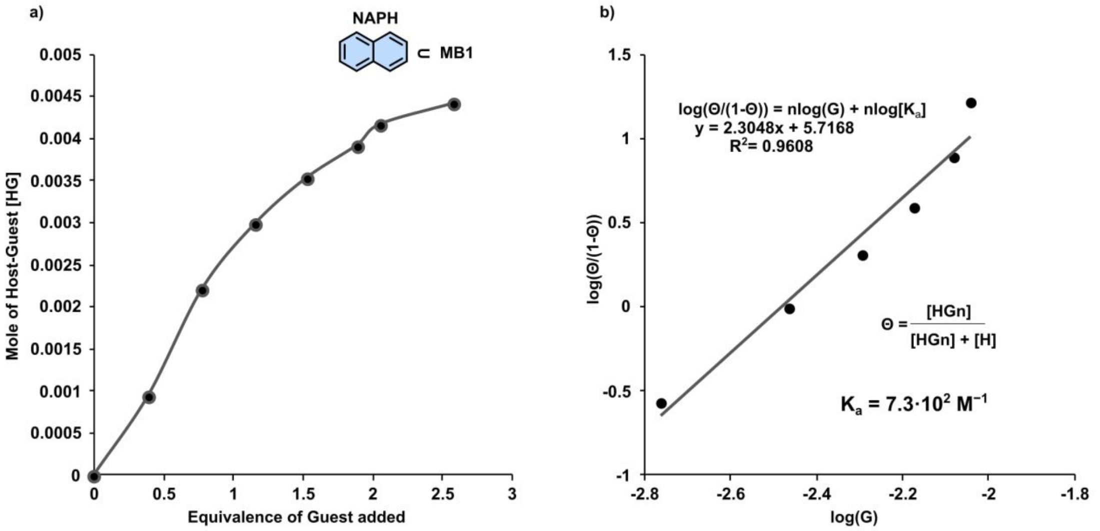

Figure S22: Titration curve of **MB1** with NAPH (Naphthalene) a) plot of Mole of Host-Guest [HG] formed vs Equivalence of Guest added b) plot of log (θ/(1−θ)) vs log[G].

$$\Delta G^{\circ} = -RT\ln(7.3\cdot 10^{2})$$

$$\Delta G^{\circ} = -16.36 \text{ kJ/mol}$$

Interpretation

Naphthalene has a more negative ΔG°, meaning stronger and more favorable binding inside the cage.

Phenanthrene binds more weakly.

$$| \Delta G^{\circ}_{PHE} | > | \Delta G^{\circ}_{NAPH} |$$

Therefore:

Naphthalene has a higher affinity for the cage than phenanthrene.

7. Selective host-guest chemistry

In a 5 mL glass vial, **MB1** (10 mg, 0.002 mmol) and an equimolar amount of the guest molecules were combined with a D_2O solution (0.5 mL) and stirred for 24 h at 60°C. The unencapsulated excess guest was subsequently removed by filtration. The resulting clear solution, containing the guest ⊂ **MB1** inclusion complex, was examined by NMR spectroscopy.

Initially, the host-guest experiments were performed in a D_2O/CD_3CN-d_3 (4:1, v/v) mixture; however, due to the increased solubility of the guest molecules in this solvent system, no selective encapsulation was observed. Therefore, D_2O was chosen as the optimal medium, in which selective host-guest complexation occurred.

To evaluate the selective host-guest behavior of **MB1**, a 1:1 molar mixture of naphthalene and phenanthrene was added to the D_2O solution of **MB1** and the mixture was stirred for 24 h at 60°C. The resulting solution was washed with $CDCl_3$ to remove any unbound or weakly associated guest molecules. Subsequently, 1,3,5-trimethoxybenzene was introduced to the aqueous phase, and the extent of guest encapsulation was determined by comparing the NMR signals of the extracted guests.

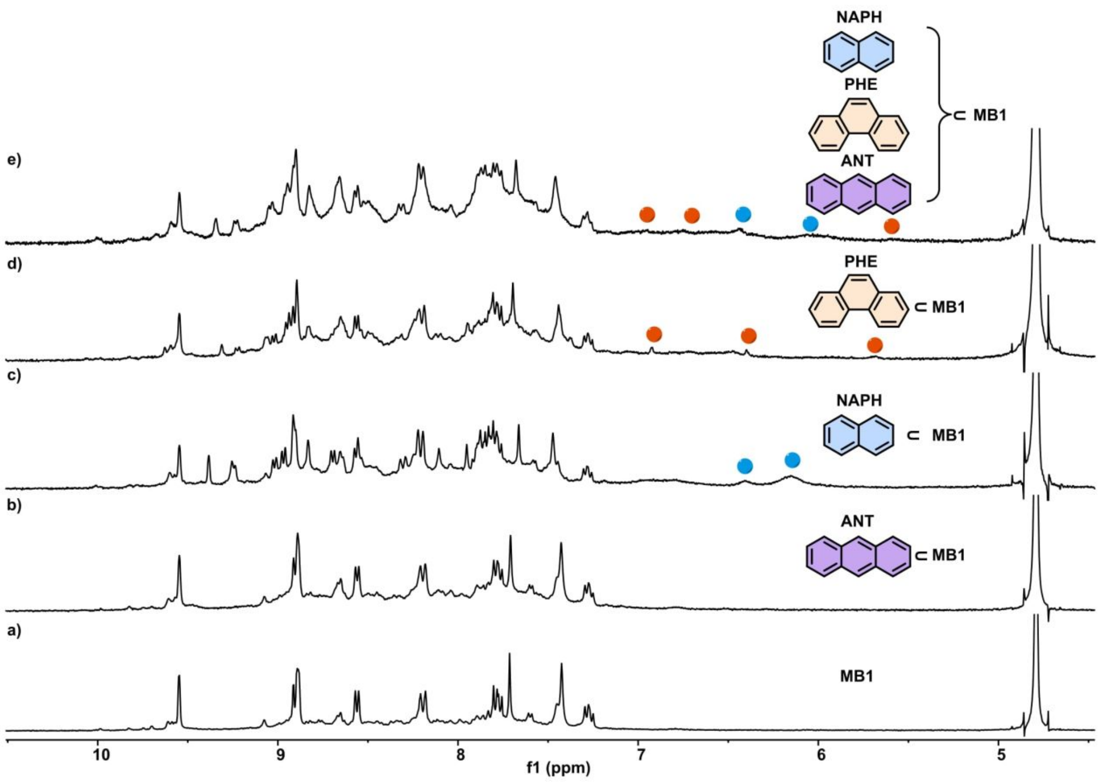

Figure S23. ^{1}H NMR (300 MHz, D_2O) stack plot of a) **MB1**, b) ANT⊂**MB1** (ANT: Anthracene), c) NAPH⊂**MB1** (NAPH: Naphthene), d) PHE⊂**MB1** (PHE: Phenanthrene), e) mixture of ANT, PHE, and NAPH (2:2:2) showing the change in NMR by guest encapsulation.

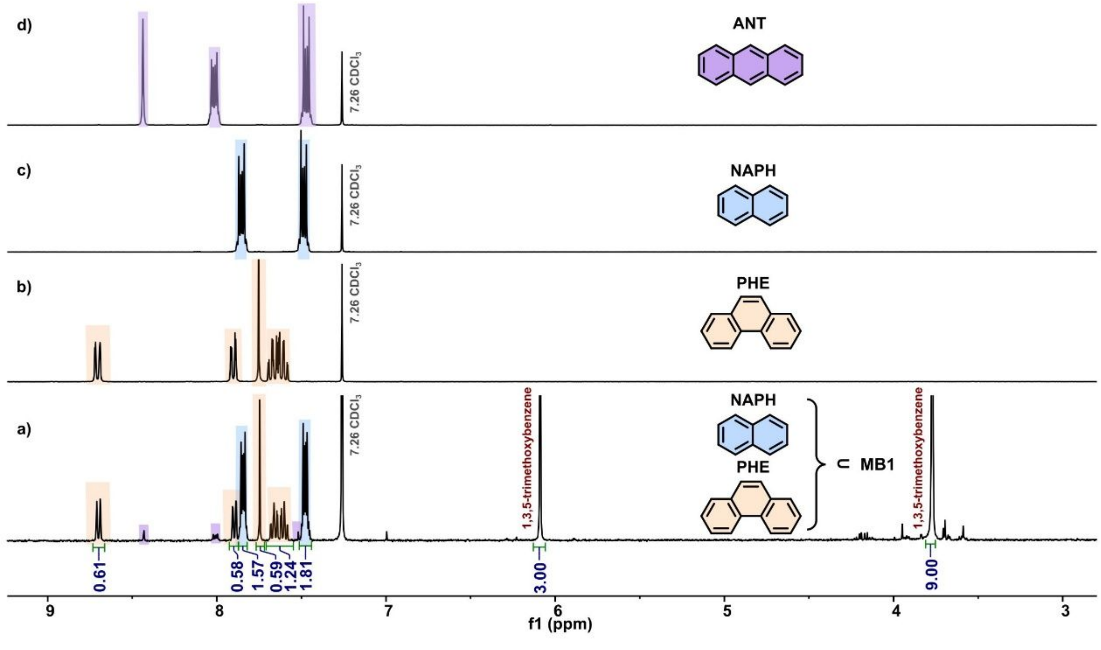

Figure S24. ^{1}H NMR (300 MHz, $CDCl_3$) stack plot of a) mixture of ANT, NAPH and PHE (2:2:2)□**MB1** (ANT: Anthracene, PHE: Phenanthrene, NAPH: Naphthene) and 1,3,5-trimethoxybenzene, b) PHE (PHE: Phenanthrene), c) NAPH (NAPH: Naphthalene), d) ANT (ANT: Anthracene). Trace amounts of anthracene are visible in the spectrum due to the formation of a suspension and/or incomplete centrifugation of the solution; however, their integral values are below 0.05 H and therefore negligible.

Based on the quantitative integrals, the ratio of naphthalene to phenanthrene in the extracted $CDCl_3$ solution is approximately 1.4 : 1. This corresponds to a composition of approximately 58% naphthalene and 42% phenanthrene in the extracted $CDCl_3$ solution.

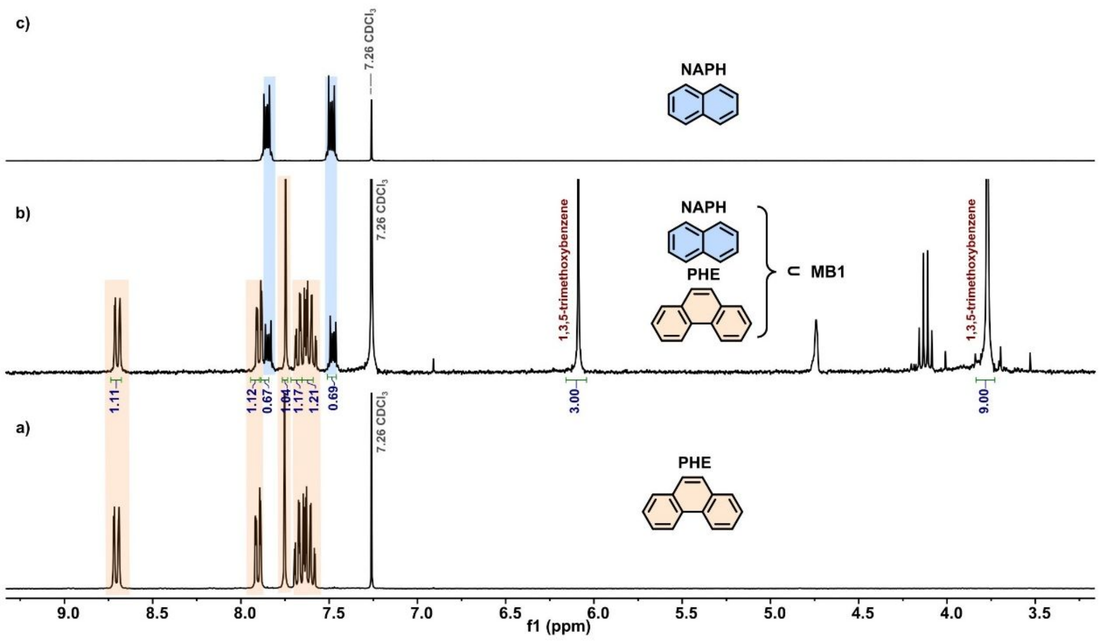

Figure S25. ^{1}H NMR (300 MHz, $CDCl_3$) stack plot of a) **PHE** (PHE: Phenanthrene), b) mixture of NAPH and PHE□**MB1** (PHE: Phenanthrene, NAPH: Naphthene) and 1,3,5-trimethoxybenzene, c) NAPH (NAPH: Naphthalene).

Accordingly, the molar ratio of naphthalene to phenanthrene in the extracted $CDCl_3$ solution is: NAPH:PHE = 1: 2.9. This corresponds to a guest composition of approximately 25.7% naphthalene and 74.3% phenanthrene (mol%).

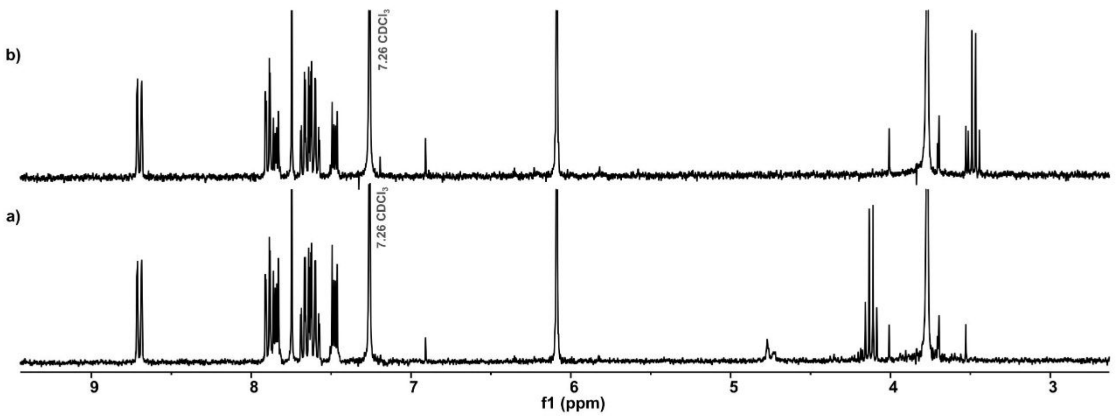

Figure S26. ^{1}H NMR (300 MHz, $CDCl_3$) stack plot of a) mixture of NAPH and PHE□**MB1** (PHE: Phenanthrene, NAPH: Naphthene) and 1,3,5-trimethoxybenzene after first extraction, c) mixture of NAPH and PHE□**MB1** (PHE: Phenanthrene, NAPH: Naphthene) and 1,3,5-trimethoxybenzene after third extraction.

8. References

(1) McPhillips, T. M.; McPhillips, S. E.; Chiu, H.-J.; Cohen, A. E.; Deacon, A. M.; Ellis, P. J.; Garman, E.; Gonzalez, A.; Sauter, N. K.; Phizackerley, R. P.; et al. Blu-Ice and the Distributed Control System: software for data acquisition and instrument control at macromolecular crystallography beamlines. *J. Synchrotron Radiat.* **2002**, *9* (6), 401-406. DOI: https://doi.org/10.1107/S0909049502015170.

(2) Cowieson, N. P.; Aragao, D.; Clift, M.; Ericsson, D. J.; Gee, C.; Harrop, S. J.; Mudie, N.; Panjikar, S.; Price, J. R.; Riboldi-Tunnicliffe, A.; et al. MX1: a bending-magnet crystallography beamline serving both chemical and macromolecular crystallography communities at the Australian Synchrotron. *J. Synchrotron Radiat.* **2015**, *22* (1), 187-190. DOI: https://doi.org/10.1107/S1600577514021717.

(3) Kabsch, W. Automatic processing of rotation diffraction data from crystals of initially unknown symmetry and cell constants. *J. Appl. Crystallogr.* **1993**, *26* (6), 795-800. DOI: https://doi.org/10.1107/S0021889893005588.

(4) Sheldrick, G. Crystal structure refinement with SHELXL. *Acta Crystallogr. Sect. C* **2015**, *71* (1), 3-8. DOI: https://doi.org/10.1107/S2053229614024218.

(5) Dolomanov, O. V.; Bourhis, L. J.; Gildea, R. J.; Howard, J. A. K.; Puschmann, H. OLEX2: a complete structure solution, refinement and analysis program. *J. Appl. Crystallogr.* **2009**, *42* (2), 339-341. DOI: https://doi.org/10.1107/S0021889808042726.

(6) Spek, A. PLATON SQUEEZE: a tool for the calculation of the disordered solvent contribution to the calculated structure factors. *Acta Crystallogr. Sect. C* **2015**, *71* (1), 9-18. DOI: https://doi.org/10.1107/S2053229614024929.

(7) Yang, D.; Greenfield, J. L.; Ronson, T. K.; von Krbek, L. K. S.; Yu, L.; Nitschke, J. R. LaIII and ZnII Cooperatively Template a Metal–Organic Capsule. *J. Am. Chem. Soc.* **2020**, *142* (47), 19856-19861. DOI: https://doi.org/10.1021/jacs.0c09991.
